# Updated nationally determined contributions collectively raise ambition levels but need strengthening further to keep Paris goals within reach

**DOI:** 10.1007/s11027-022-10008-7

**Published:** 2022-06-21

**Authors:** Michel G. J. den Elzen, Ioannis Dafnomilis, Nicklas Forsell, Panagiotis Fragkos, Kostas Fragkiadakis, Niklas Höhne, Takeshi Kuramochi, Leonardo Nascimento, Mark Roelfsema, Heleen van Soest, Frank Sperling

**Affiliations:** 1 PBL Netherlands Environmental Assessment Agency, PO Box 30314, 2500 GH, The Hague, The Netherlands; 2 Institute for Environmental Studies (IVM), Vrije Universiteit Amsterdam, Amsterdam, The Netherlands; 3 International Institute for Applied Systems Analysis (IIASA), Schlossplatz 1, 2361, Laxenburg, Austria; 4 E3-Modelling S.A, Panormou 70-72, Athens, Greece; 5 NewClimate Institute, Cologne, Germany; 6 Environmental Systems Analysis Group, Wageningen University, Wageningen, The Netherlands; 7 Copernicus Institute of Sustainable Development, Utrecht University, Princetonlaan 8a, 3584 CB, Utrecht, The Netherlands

**Keywords:** Climate change mitigation, Climate policy, NDC, Greenhouse gas emissions scenario, Paris Agreement, Integrated assessment models

## Abstract

By January 2022, 156 countries had submitted new or updated nationally determined contributions (NDCs) under the Paris Agreement. This study analyses the greenhouse gas (GHG) emissions and macroeconomic impacts of the new NDCs. The total impact of the updated unconditional and conditional NDCs of these countries on global emission levels by 2030 is an additional reduction of about 3.8 and 3.9 GtCO_2_eq, respectively, compared to the previously submitted NDCs as of October 2020. However, this total reduction must be about three times greater to be consistent with keeping global temperature increase to well below 2 °C, and even seven times greater for 1.5 °C. Nine G20 economies have pledged stronger emission reduction targets for 2030 in their updated NDCs, leading to additional aggregated GHG emission reductions of about 3.3 GtCO_2_eq, compared to those in the previous NDCs. The socio-economic impacts of the updated NDCs are limited in major economies and largely depend on the emission reduction effort included in the NDCs. However, two G20 economies have submitted new targets that will lead to an increase in emissions of about 0.3 GtCO_2_eq, compared to their previous NDCs. The updated NDCs of non-G20 economies contain further net reductions. We conclude that countries should strongly increase the ambition levels of their updated NDC submissions to keep the climate goals of the Paris Agreement within reach.

**Supplementary Information:**

The online version contains supplementary material available at 10.1007/s11027-022-10008-7.

## Introduction

In the Paris Agreement under the United Nations Framework Convention on Climate Change (UNFCCC), governments agreed to the long-term target of keeping the increase in global average temperature to well below 2 °C above pre-industrial levels and to pursue efforts to limit the temperature increase to 1.5 °C (UNFCCC 2015). Before and during the Paris conference, countries submitted comprehensive NDCs to the UNFCCC that outlined their self-determined post-2020 targets. This previous round of submitted NDCs would deliver only half of the emission reductions needed by 2030 relative to a no policy baseline compared to what is necessary to stay on a global least-cost pathway towards keeping warming levels well below 2 °C (Höhne et al. 2020; Rogelj et al. 2016).

Under the Paris Agreement, countries agreed to periodically update their contributions, over time, by an iterative process in 5-year intervals, informed by reviews of the status of contributions (global stocktake) (UNFCCC 2015). Countries were expected to formulate and submit their updated NDCs and long-term development strategies well in advance of COP26 in 2021. By the end of January 2022, 156 countries (including 27 EU Member States) have updated their NDC submissions (ClimateWatch 2022; UNFCCC 2022). These countries represent about 84% of global GHG emissions in 2019, including emissions and removals from land use, land-use change and forestry (LULUCF) (FAOSTAT 2020; Olivier and Peters 2020).

Building on an earlier study (den Elzen et al. 2016), we analysed the GHG emissions and macroeconomic impacts of the second round of NDC submissions on both the global level and the G20 economies, including the European Union-27 (EU-27).^1^ More specifically, we analysed the likely impact of fully implemented NDCs of 143 countries^2^ (representing about 93% of global emissions in 2019, excluding the international bunker emissions) on global GHG emission levels up to 2030 (Supplementary Text 2), and the additional reductions resulting from the new or updated NDCs, compared to the projections based on the previous NDCs (October 2020).^3^ Section 2 presents the NDCs of the G20 economies. Section 3 describes the used methodology. In Section 4, results are compared to emission projections based on current implemented policies and to projected global emission levels that need to be achieved by 2030 according to least-cost emission scenarios to stay on track to meet the climate goals of the Paris Agreement. Section 5 compares the NDC targets against GHG emission projections from current domestic climate policies up to 2030. In addition, it shows the impact NDCs of the G20 countries in terms of different important emission indicators, comparing the ambition levels of these countries. Section 6 analyses the impact of emissions and removals from LULUCF on achieving the NDCs. Finally, Section 7 analyses the macroeconomic impacts of the new or updated NDCs for 2030, and Section 8 concludes.

## Overview of new NDC submissions of the G20 economies

The European Union has submitted a more stringent NDC target, aiming for a reduction in domestic net GHG emissions of at least 55% below 1990 levels, by 2030 (Table 1). This target has been secured by legislation as part of the European Climate Law, and proposals for measures to achieve the target were published under the Fit-For-55 policy package in July 2021 (European Commission 2021). At the US Leaders Summit on Climate on 22 April 2021, the US Government presented its updated NDC, including a net GHG emission target of 50–52% below 2005 levels, by 2030. In October 2021, China submitted to the UNFCC its updated NDC, which is an enhancement compared to the previous. China intends to peak CO_2_ emissions before 2030 and lower its carbon dioxide emissions per unit of GDP by over 65% from 2005 levels, by 2030. Additionally, by 2030, China intends to increase the share of non-fossil fuel energy in primary energy consumption to around 25% and total wind and solar generation capacity to over 1200 gigawatts. Two G20 economies (India and Turkey) had not officially revised their NDC targets, whereas Australia resubmitted their first NDC targets in 2021 for the second time. In November 2021, Brazil announced a target to reduce 2030 emissions by 50% but failed to specify the reference for this reduction. This target is not considered in this research since it was not officially submitted to the UNFCCC. Here, we consider the Brazilian target to reduce emissions by 43% below 2005 values by 2030. Table 1 presents an overview of the previous and updated NDC submissions of all G20 economies, representing three-quarters of global GHG emissions in 2019 (for all countries, see Supplementary Text 1 and Text 2) (FAOSTAT 2020; Olivier and Peters 2020).

**Table 1:** Summary of targets of the new or updated NDCs by the G20 economies (except the EU Member States), as of 31 January 2022 (UNFCCC 2022), compared to the previous NDCs, as of October 2020 (UNFCCC 2022). All target values are for 2030, unless otherwise specified

Country	Previous NDCs	New or updated NDCs	Notes
Argentina	Cap 2030 net emissions at 483 MtCO_2_eq (unconditional) and 369 MtCO_2_eq (conditional)	Net emissions cap at 349 MtCO_2_eq in 2030 (unconditional)	
Australia	Reduce GHG emissions by 26–28% from 2005 levels	Reduce GHG emissions by 26–28% from 2005 levels. Updated NDC to be implemented as an emission budget	
Brazil	Reduce GHG emissions by 37% from 2005 levels by 2025 and (indicatively) 43% from 2005 levels	Reduce GHG emissions by 43% from 2005 levels by 2030	Increased base year emissions
Canada	Reduce GHG emissions by 30% from 2005 levels	Reduce GHG emissions by 40–45% from 2005 levels	
China	- Peak CO_2_ emissions around 2030 - Reduce CO_2_/GDP by 60–65% from 2005 levels - Share of non-fossil fuels in primary energy consumption to around 20% - Increase forest stock volume by around 4.5 billion cubic metres	- Peak CO_2_ emissions before 2030 - Reduce CO_2_/GDP by 65% from 2005 levels - Share of non-fossil fuels in primary energy consumption to 25% - Increase forest stock volume by around 6 billion cubic metres - Increase the installed capacity of wind and solar power to 1,200 GW	
EU-27	Reduce GHG emissions by at least 40% from 1990 levels (for the EU-28)	Reduce net GHG emissions by at least 55% from 1990 levels^i^	
India	- Reduce emissions/GDP by 33–35% from 2005 levels - To achieve about 40% cumulative electric power installed capacity from non-fossil fuel based energy resources by 2030 (Conditional)	Not available (N.A.)	
Indonesia	Reduce GHG emissions by 26% (unconditional) and 29% (conditional) relative to business-as-usual (BAU)	Reduce GHG emissions by 26% (unconditional) and 29% (conditional) relative to BAU	
Japan	Reduce GHG emissions by 26% from 2013 levels (fiscal year)	Reduce GHG emissions by 46% from 2013 levels (fiscal year)	
Mexico	Reduce GHG emissions by 22% (unconditional) and 36% (conditional) from BAU	Reduce GHG emissions by 22% (unconditional) and 36% (conditional) from BAU	Increased BAU
Russian Federation	Limit 2030 emissions to 70–75% of 1990 level^ii^	Limit 2030 emissions to 70% of 1990 level^ii^	
Saudi Arabia	Annually abate up to 130 MtCO_2_eq by 2030	Annually abate up to 278 MtCO_2_eq by 2030	
South Africa	Limit 2025–2030 emissions to a range between 398 and 614 MtCO_2_eq	Limit 2025–2030 emissions to a range between 350 and 420 MtCO_2_eq	
Republic of Korea	Reduce GHG emissions by 37% from BAU	Reduce GHG emissions by 40% from 2018 levels	
Turkey	Reduce GHG emissions by up to 21% from BAU	N.A	
UK	(Part of EU-28)	Reduce GHG emissions by at least 68% from 1990 levels	
USA	Reduce GHG emissions by 26–28% from 2005 levels by 2025	Reduce GHG emissions by 50–52% from 2005 levels by 2030	

^i^The new target is net GHG reduction (emissions after deduction for removed emissions must be 55% below their 1990 level). The former target included emissions from LULUCF, but not in the headline target — only the requirement that Member States balance emissions and their removal as per the LULUCF regulation. ^ii^Taking into account the maximum possible absorptive capacity of forests

## Methods

Based on the new and updated NDC submissions, this study presents GHG emissions in CO_2_ equivalent terms,^4^ both in terms of excluding and including the LULUCF sector. In this section, we outline how the emission projections for the current policies and NDC scenarios were derived, and how contributions of countries from the LULUCF sector to mitigation ambitions were accounted for. This is followed by a description of the methodological approach for comparing the emission projections from the NDC commitments with current policies scenarios and assessing the macroeconomic implications of NDCs.

### Overview of the scenarios

This study considers the following seven different scenarios.

#### Current policies scenario

The impact of implemented policies on GHG emissions in all sectors up to 2030 has been projected using the integrated assessment model IMAGE (Stehfest et al. 2014), which includes the TIMER energy system model (see Appendix A1–A.2). The starting point for the calculations of the impact of climate policies is the updated SSP2 (no climate policy) reference scenario, as implemented with the IMAGE model (Roelfsema et al. 2020; Van Vuuren et al. 2017b). Current climate and energy policies from G20 economies, as identified in the public database on climate policies (Nascimento et al. 2022),^5^ the ENGAGE project and policy overview updates (Kuramochi et al. 2019; 2021), were added on top of this IMAGE SSP2 reference scenario (Roelfsema et al. 2022; 2020) (Supplementary Text 3).^6^ The current policies scenarios also take into account the short-term (2020–2025) economic projections that were updated to include the implications of the COVID-19 pandemic, including changes in sectoral activity (Dafnomilis et al. 2021).

For 12 major emitting non-G20 countries,^7^ the IMAGE calculations are supplemented with current policies scenario projections from Kuramochi et al. (2021) with updates from Nascimento et al. (2021). Both used baseline scenarios published by national governments and international organisations as a starting point. The LULUCF CO_2_ emissions and their removals were calculated using the global GLOBIOM and G4M land-use models (Havlík et al. 2014) (see Appendix A.3). The starting point for the climate policy LULUCF calculations is the latest SSP2 reference scenario as implemented in the GLOBIOM and G4M models (Fricko et al. 2017). Current LULUCF policies for major emitting countries were added to that baseline (Nascimento et al. 2021).

#### Unconditional and conditional updated NDC scenario

The NDC scenario assumes full implementation of NDC targets, using the updated NDCs if available (as of 31 January 2022). Several countries have distinguished unconditional and conditional targets^8^ in their NDCs, which is why we have introduced both an unconditional and conditional NDC scenario. For countries whose NDCs include unconditional targets only, emission levels are assumed to be the same in both scenarios. For countries having only conditional NDC targets, unconditional NDC emission levels were assumed to equal those from the current policies scenario.

#### Unconditional and conditional previous NDC scenario

The same scenarios as the updated NDC scenarios as described above, but here assuming a full implementation of the earlier NDC submissions, as of October 2020 (UNFCCC 2022).

#### 2 °C and 1.5 °C scenarios

The 2 °C scenario represents the long-term least-cost emissions pathway consistent with holding global warming below 2 °C throughout the twenty-first century with at least 66% chance. The 1.5 °C scenario represents least-cost pathway consistent with holding global warming below 1.5 °C throughout the twenty-first century with limited or no overshooting. Global warming in 2100 is projected to be below 1.5 °C with at least 66% chance, while throughout the twenty-first century it is kept below 1.5 °C with at least 33% chance. The global emissions pathways for the 2 °C and 1.5 °C scenarios are based on UNEP (2019), which were calculated from scenarios underlying the IPCC Special Report on Global Warming of 1.5 °C (IPCC SR1.5) (Rogelj et al. 2018).

### Historical emissions

The GHG emissions projections for the current policies and NDC scenarios were harmonised with historical 1990–2019 emissions, i.e. adding the absolute emissions difference in the harmonisation year between the inventory data and the model data to the model projections until 2030. For most Annex I countries, historical emissions data is based on the 2019 Greenhouse Gas Inventories submitted to the UNFCCC (2021a). If available, for non-Annex I countries, the historical data was taken from the national reports (National Communications and Biennial Update Reports) (UNFCCC 2021a, b, d), otherwise EDGAR database (Olivier and Peters 2020) and FAOSTAT (2020) (LULUCF emissions) (for details for the major emitting countries, see Nascimento et al. (2021)). The harmonisation year was 2019 for Annex I countries and the latest data year for non-Annex I countries.

### Emission projections (excluding LULUCF) for NDCs

This section provides the methodology used for calculating the emission projections (excluding the emissions and removals from LULUCF) for NDC targets of the G20 and non-G20 economies. The G20 countries, together account for about 75% of global GHG emissions in 2019 based on the EDGAR database (GHG emissions excluding those from LULUCF) (Olivier et al. 2020) and FAOSTAT (2020) (LULUCF emissions). Therefore, their NDCs were analysed in more detail than those from non-G20 countries.

#### G20 economies

The NDC emission targets by 2030 in absolute terms are based on the NDC submissions (UNFCCC 2022). In case the target could not directly be obtained from the NDC submissions, national inventories or communications were used to derive historical emissions data or business-as-usual (BAU) projections, if necessary. More specifically, national inventories were used to calculate the absolute NDC emission targets of Australia, Brazil, Canada, the EU-27 and the UK, Japan, the Republic of Korea, the Russian Federation and the USA. The absolute emission targets were directly calculated from the NDC submissions for Argentina, Indonesia, Mexico and Turkey. Supplementary Text 4 shows how we quantified the mitigation components of the previous NDCs and the updated or new NDCs, submitted by G20 members. This includes emission reduction targets, base years to which targets are defined, and calculated absolute emission targets for 2030.

For China and India, the quantification of NDC target emission levels is more complicated because they comprise a combination of targets that include non-fossil energy targets, timing of CO_2_ emissions peak (China only), forest targets, and emission intensity reduction targets. Therefore, calculated emissions highly depend on the assumptions applied. The starting point for the calculations for China is the current policies scenario. The following targets of the original NDC and updated NDC are all already achieved in our current policies scenario: the peak of CO_2_ emissions is before 2030, the reduction of CO_2_ intensity reaches at least 65% by 2030, the increase of the Chinese forest stock volume by 6 billion m^3^, and the non-fossil share in energy consumption reaches at least 25% by 2030. However, the pledged increase in wind and solar capacity of 1200 GW, as included in the updated NDC, is not reached (800 GW is achieved in the current policies scenario). The impact of the pledged wind and solar capacity targets compared to the current policies scenario is about − 0.8 GtCO_2_eq in 2030 as calculated using the TIMER energy system model. Given these considerations, in this study, the current policies scenario is assumed to be equal to the previous NDC scenario, which is consistent with the scenarios developed by two national models for China (PECE V2.0 and IPAC), as presented in Fragkos et al. (2021b) and the COMMIT scenario database (van Soest et al. 2021a). For India, we calculated the combined effect of intensity targets and the non-fossil target using the TIMER energy model of IMAGE (den Elzen et al. 2016). For the unconditional NDC scenario, the emissions intensity target is assumed to apply to total GHG emissions excluding agricultural and LULUCF emissions, and if adding non-mitigated agricultural and LULUCF emissions, projected GHG emission levels are 4.3–4.5 GtCO_2_eq in 2030. For the conditional NDC scenario, the implementation of the renewable target in the TIMER model projects about 0.3 GtCO_2_eq additional reductions by 2030 compared to the unconditional scenario.

For the USA, the impact of the updated NDC of − 0.85 GtCO_2_eq is calculated relative to the estimated 2030 emission levels, which were linearly interpolated between the 2025 emission target (previous NDC) and the 2050 national long-term target (83% below 2005 levels by 2050).

#### Non-G20 economies

A total of 99 non-G20 countries were covered in the NDC analysis (18% of emissions by 2019). For 25 non-G20 countries, the NDCs are defined by reduction targets relative to a historical base year and can be translated into absolute levels in a straightforward manner (see Supplementary Text 2). For about 60 countries, NDCs are defined as reduction target relative to business-as-usual (BAU) emission projections, which is generally included in the NDC submissions (see UNFCCC website). Four countries provide intensity targets, and the remaining countries have NDCs defined as reduction actions for land-use emissions (Section 3.3). The emission levels of countries that are not included in the analysis follow the downscaled IMAGE current policies scenario. For 12 major emitting non-G20 countries,^9^ the estimation of NDC target emission levels was based on the Climate Action Tracker project (Climate Action Tracker 2021), as described by Nascimento et al. (2021).

#### Global emissions

For the calculation of the global GHG emissions estimate for the NDC scenarios, we assume that if countries are overachieving their NDC targets under current policies, the emission level of the current policies is used. For both the NDC scenarios and current policies scenario, total global emissions were calculated by adding to the total emission levels of the G20 and non-G20 economies: (i) emissions from international aviation and international shipping, which, together, is about 1.7 GtCO_2_eq by 2030, based on the current policies scenario of IEA (2019); and (ii) calculated remaining LULUCF emissions based on various sources (including FAOSTAT, national communication and model projections) for the countries which NDC did not address LULUCF, which is together about 0.8 GtCO_2_eq by 2030 (Section 3.4).

### LULUCF emission projections in the NDCs

Most of the G20 economies report emission target levels that include emissions and their removals related to mitigation options in the LULUCF sector, such as afforestation and reforestation. Although there are uncertainties concerning the accounting approaches and methodologies used by countries to account for LULUCF-related emissions and emission removal, we assume that all countries, unless otherwise stated in their NDC, will apply the net–net accounting approach for all carbon pools and LULUCF sub-sectors. This means that emissions from land use are included in the same way as those from the other sectors (see Supplementary Text 5 and Table S.3).

NDC scenario projections of LULUCF emissions and their removal were developed based on the NDCs that were submitted (UNFCCC 2022). In cases where no quantitative information on LULUCF sector developments in line with NDC targets could be derived, we analysed Biennial Update Reports (BUR) and national supporting documents. Thereafter, the projections were compared to the projections of LULUCF-related emissions developed based on the previous NDCs as documented by Forsell et al. (2016), to evaluate which countries have pledged higher emission reduction targets for the LULUCF sector. In terms of the G20 countries, NDC scenario projections for Argentina, Japan and Mexico were derived from their updated NDC submissions (UNFCCC 2022). For Australia, Canada and the EU-27, projections were derived from supporting documents (European Commission 2020; UNFCCC 2021c). In case LULUCF projections could not be derived from updated NDCs, nor from support documents, we used projections from Forsell et al. (2016). The resulting LULUCF emissions and their removal in 2030, according to the NDC scenarios, are shown in Table S.5. Supplementary Text 7 provides further information concerning the NDCs; the scenario projections were harmonised with the emission projections excluding LULUCF.

### Macroeconomic impacts

The implementation of climate policies and NDCs affect the economy in multiple ways, as they impact the demand for goods and services, sectoral production, labour markets, income levels and bilateral trade. We used the Computable General Equilibrium model GEM-E3-FIT (Paroussos et al. 2020) to quantify macroeconomic, trade, employment and distributional impacts of the new NDCs of G20 economies (see Appendix A.4). For this purpose, we included the calculated NDC targets for G20 economies (in the form of specific emission targets or emission-intensity targets for 2030 (excluding the emissions and removals from LULUCF)) in GEM-E3 that act as constraints in model equations and lead to increased economy-wide regional and country-specific carbon prices, depending on the NDC ambition level. In the current study, we assume that carbon revenues in GEM-E3-FIT are recycled through the public budget, in order to increase government savings and thus support investment (which are required for the clean energy transition). Other carbon revenue recycling schemes can also be implemented in GEM-E3-FIT, e.g. lump-sum transfer to households and reduce social security contributions (as in Fragkos et al. 2021a).

Carbon prices represent a general metric for the intensity of CO_2_ reduction policies. GEM-E3 uses the same NDC emission reduction targets (relative to 2015 levels, all GHG emissions excluding LULUCF) up to 2030 (see Supplementary Text 7, Table S.5), as described in Section 3.3, ensuring consistency with the emission calculations presented in Table 1. The GEM-E3 model also has a current policies scenario. To ensure full consistency of current policies implementation, the IMAGE model and GEM-E3 have assumed the same climate and energy policies in terms of modelling protocol for the implementation (Nascimento et al. 2021).^10^

## Impact of NDCs on global and national emissions

### Impact of new NDCs on global emissions compared with previous NDCs

**Fig. 1:**
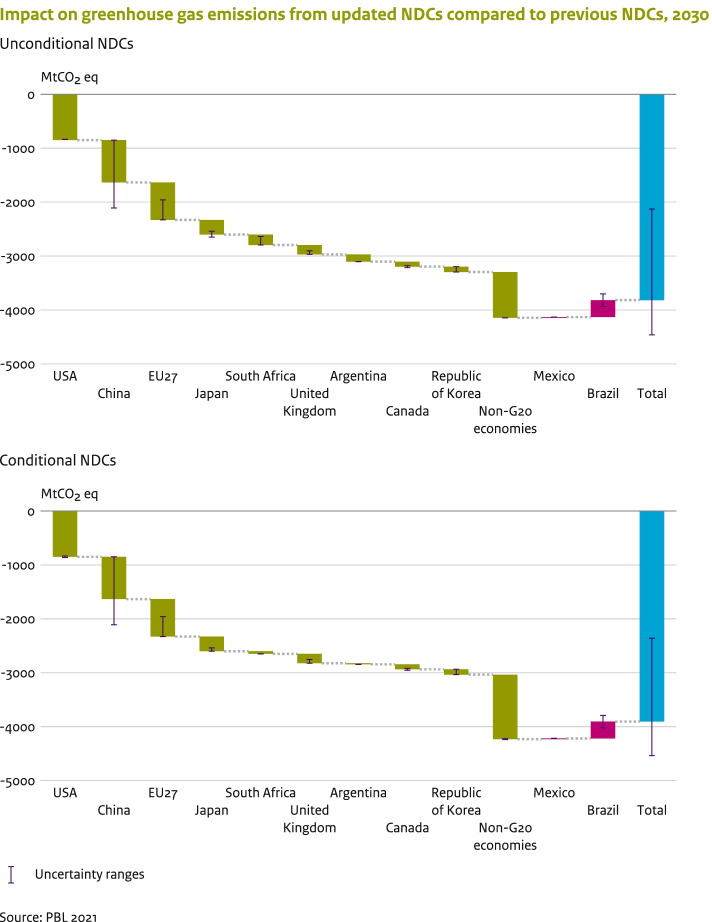
The contribution of the updated NDC targets of countries on total additional global emission reduction (as indicated in blue column) based on the full implementation of the *unconditional* NDC targets (upper figure) and *conditional* NDC targets (lower figure), compared to the previous NDCs. The green columns indicate additional reductions from individual G20 economies, the non-G20 countries as a group with their stronger NDC targets (compared to their previous NDCs), and the red columns indicate the increase in emissions from G20 economies with their weaker NDC targets. The uncertainty ranges represent the reductions relative to the current policies scenarios (such as for the EU-27), or other uncertainties related to the range in reduction targets (such as South Africa), or the uncertainties in the base-year emissions (Brazil). Some G20 economies are not shown as they have submitted the same reduction targets in their updated NDC or have not yet submitted an updated NDC (see Table 1)

The full implementation of all unconditional updated NDC targets is estimated to have an aggregated reduction impact on global GHG emissions of about 3.8 (2.1–4.5) GtCO_2_eq by 2030, compared to that of the previous NDCs (Fig. 1). If the conditional updated NDCs are also fully implemented, the total reduction impact would be 3.9 (2.4–4.5) GtCO_2_eq, so about 0.1 GtCO_2_eq higher (Fig. 1).

Nine G20 economies with updated NDCs have pledged more stringent GHG reduction targets by 2030, leading to total additional reductions of 3.3 (1.7–3.8) GtCO_2_eq, compared to those in the previous NDCs (Fig. 1). The largest additional contributions come from the USA, China, the EU-27, Japan, South Africa and the UK. For China, the impact of the climate targets of the updated NDC is calculated relative to the current policies projections, accounting for recently adopted policies. The impact of the NDC is a reduction of 0.8 (0–1.2) GtCO_2_eq relative to current policies projection of about 14.5 (12.1–14.5) GtCO_2_eq,^11^ which is due to the impact of the increase in the installed capacity of wind and solar power, as the other updated NDC targets are met under the current policies (Section 3.1). The uncertainty range represents the uncertainty around the implementation of the additional renewable capacity.

There are also some non-G20 economies with new NDC targets with more stringent reduction targets (higher coverage of sectors and larger reduction targets). Together these new targets result on a total impact of 0.85 GtCO_2_eq, with major contributions from Chile, Colombia, Nigeria and Ukraine, that present stronger reductions targets and/or reduced BAU emission projections for 2030. For the conditional NDCs, the impact would be 0.3 GtCO_2_eq higher.

The enhanced ambition level for the unconditional NDCs targets leads to projected aggregated emission reductions in global emissions of about 3.8 (2.1–4.5) GtCO_2_eq (see Fig. 1). We see similar estimates for the conditional NDCs. However, about 0.35 (0.2–0.5) GtCO_2_eq of the additional emission reduction is counterbalanced by the higher emissions from a group of countries with lower ambition levels than in their previous NDCs. There are two reasons for higher emission levels: (1) changes in the historical reference period (e.g. Brazil) and (2) changes in the projected BAU baseline. More specifically:

1. For Brazil, the updated NDC leads to an absolute increase in emissions. The updated NDC presents the same relative reduction target in percentage, i.e. reduction of 37% and 43% by 2025 and 2030, respectively, compared to 2005 levels. However, the assessment metric of the updated NDC was revised. While the 1st NDC refers to the Second National Inventory Report (NIR), the updated NDC follows the Third NIR (AR5 metrics). Since about two-thirds of Brazil’s emissions are related to land use, the updated methane GWP (from 21 to 34) has a large impact on emissions. Thus, the baseline year emissions move from 2.1 to 2.8 GtCO_2_eq,^12^ which increases the updated emission target in 2030 from 1.2 to 1.6 GtCO_2_eq.
2. For Mexico, the projected BAU baseline emissions have increased, whereas the reduction targets have not changed, which increases the emission target slightly. In some other countries, such as Cambodia and Zambia, baseline emissions in the updated NDC submission have increased, as BAU emissions of the NDCs of 2016 did not include all sources, which leads to an increase of 0.1 GtCO_2_eq, which is accounted for the net reduction in all non-G20 economies.

The remaining G20 economies have NDC targets which lead to no additional reduction. The Russian Federation’s updated NDC did not strengthen the country’s 2030 emission target. The target emission level remains higher than current policies projections and today’s emission level. Australia and Indonesia have submitted the same reduction targets in their updated NDC (not shown in Fig. 1). A number of G20 economies still have not submitted their new or updated NDC (Table 1).

### Emission reduction projections of new NDCs compared with current policies

**Fig. 2:**
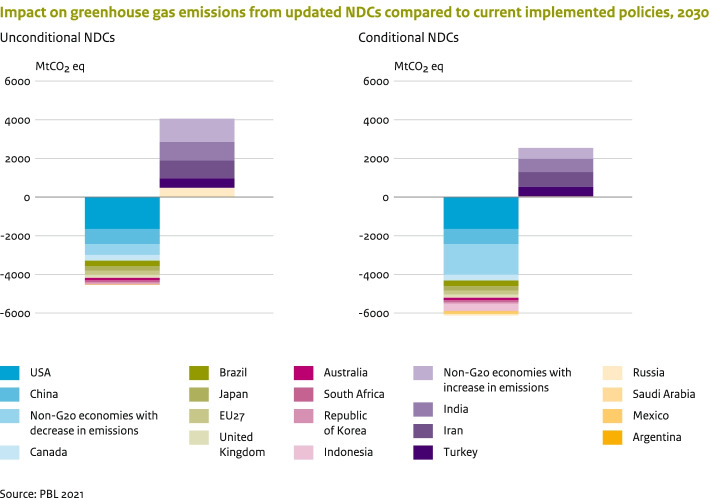
Projected absolute emission reductions relative to the current policies scenario in 2030 by the G20 countries and the non-G20 countries as a group based on their NDCs. For countries with a reduction target range, projected reductions were based on the average of the range

For the G20 economies, this section compares the NDC targets with GHG emission projections under current domestic climate policies up to 2030. The comparison with current domestic mitigation policies allows an assessment of the additional emission reductions needed to achieve the NDCs’ reduction targets. Figure 2 shows that, collectively, countries will likely need to implement additional or more stringent policies to further reduce global GHG emissions by about 4.5 GtCO_2_eq to achieve the unconditional NDCs by 2030, and by about 6.1 GtCO_2_eq to achieve the conditional NDCs. Only six economies are responsible for the largest share (about 75% for the unconditional NDCs and about 60% for the conditional NDCs) of the required reductions, namely the USA, China, Canada, EU-27, Japan and Brazil.

The emission target levels of under the NDC scenario for several countries (among which India, Russian Federation, Saudi Arabia, Turkey and several non-G20 members, such as Iran) are projected to be above the estimated current policies scenario levels.^13^ These countries are expected to overachieve their NDC targets with current policies (Fig. 2, upper part). In our assessment, we assumed that these countries follow their current policies emission trajectory and overachieve their NDCs, leading to additional net reductions at the global level of 4.1 and 2.5 GtCO_2_eq for the unconditional and conditional NDC scenario, respectively.

### Projected global GHG emission levels by 2030 if all NDCs are implemented

The resulting global GHG emission level in 2015 based on all data sources is estimated to be about 47.5 GtCO_2_eq, which is about 3.1 GtCO_2_eq below the 2015 emission estimate from EDGAR and FAO. It is also about 3.6 GtCO_2_eq below the 2015 emissions for the global least-cost emission scenarios limiting global warming to below 2 °C and 1.5 °C (UNEP 2019). Grassi et al. (2017; 2021) find an around 4 GtCO_2_eq/year difference in global LULUCF net emissions between country reports and scenarios studies (as reflected in IPCC report) mainly due to different definition of LULUCF emissions related to the ‘anthropogenic forest sink’. This identified discrepancy can be accounted and corrected for, and solutions have been analysed in Grassi et al. (2021). The analysis presented here corrects for the discrepancy in reported emissions between national GHG inventories and global emission pathway studies by applying a constant adjustment term of 3.7 GtCO_2_eq over the 2010–2030 period.

**Fig. 3:**
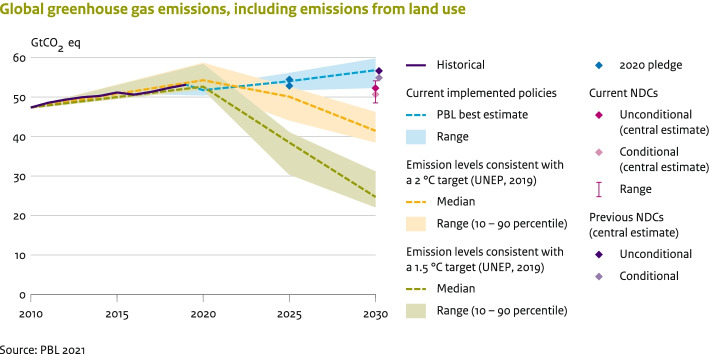
Impact of the implementation of the NDCs and current policies on greenhouse gas emission projections and on narrowing the emission gap in 2030

Global emissions are projected to peak by 2025, and reach about 3% above 2015 levels by 2030 if the unconditional NDCs are implemented, and return to 2015 levels if the conditional NDCs are implemented (see Fig. 3). This corresponds to about 11% and 7% above 2010 levels.^14^ This is well below the growth projected in the current policies scenario (11%). The projected global emission level in 2030 is about 52.3 (50.1–54.1) GtCO_2_eq if all unconditional NDCs are implemented, and 50.7 (48.5–52.2) GtCO_2_eq if the conditional NDCs are implemented (Supplementary Text 6, Table S.4). The global GHG emissions of the conditional and unconditional updated NDC scenario are about − 4.4 and − 4.2 GtCO_2_eq by 2030, respectively, compared to the previous NDCs scenario (see Fig. 3). This effect is higher than the additional reduction due to the updated NDCs alone due to the lower emission projections from countries that overachieve their NDC targets.

Our global emission projections for the NDC scenarios are surrounded with uncertainties. The largest uncertainty is related to the implementation of the mitigation targets of China and India, including the emission intensity targets, peaking ambition and non-fossil targets, and the final projected emission target levels strongly depend on future GDP trends, which are subject to large uncertainties (for further details, see Supplementary Text 4 and Text 6). The presented ranges try to quantify the above uncertainties.

### What are the emission implications for staying well below 2 °C and 1.5 °C?

This section focuses on the global impact of NDCs and compares the resulting emission level with the level needed to meet the Paris climate goals. Global emission levels consistent with a 66% chance of staying below 2 °C and 1.5 °C are projected to be 41.5 (38.7–46.0) GtCO_2_eq and 24.7 (22.3–30.9) GtCO_2_eq, respectively, for the year 2030 (median and 10th–90th percentile range). The full implementation of the unconditional NDCs is estimated to result in a gap in 2030 of 10.8 (4.1–15.4) GtCO_2_eq compared to least-cost 2 °C pathways. The emission gap between unconditional NDCs and below 1.5 °C pathways is about 27.6 (19.2–31.8) GtCO_2_eq (Fig. 3). According to our projections, this gap will be 1.5 GtCO_2_eq smaller if the conditional NDCs are implemented. The total impact of the conditional and unconditional NDC updates (3.9 and 3.8 GtCO_2_eq, respectively) has reduced the 2030 gap from current policies to 2 °C by only about 35–40% and to 1.5 °C by only 14%.

## Comparison of indicators between the G20 countries

This section looks in more detail at country results regarding the effect of NDCs on the timing and level of GHG emission peaks, per capita emissions, and whether the NDCs are in line with 1.5 °C and 2 °C.

### Timing and level of national GHG emission peaks

Full implementation of post-2020 NDCs is projected to lead to different national emission trajectories and emission peak years (Table 2). Among the G20 economies, the EU-27 was the first region where emissions peaked (around 1980). Before 1990, emissions in the Russian Federation and in the UK peaked.^15^ By 2010, half of the G20 members (EU-27, Russia, Argentina, Australia, Brazil, Canada, the UK and the USA) had peaked emissions. Emissions in Australia, Canada and the USA peaked with per capita emissions that were a factor two higher than the EU-27. By around 2013, Japan had peaked. Another three G20 members’ emissions are projected to peak by around 2020 (Republic of Korea) or before 2030 (South Africa and China for CO_2_ only) if NDCs are fully implemented. Five G20 members’ GHG emissions show no sign of peaking, given the full implementation of the unconditional NDC targets (India, Indonesia, Mexico, Saudi Arabia and Turkey).

**Table 2:** Overview of G20 member status and progress, including on Cancun pledges and NDC targets, based on UNEP (2018)

Country	Share of global GHG emissions in 2019^1)^	Projected per capita GHG emissions in 2030 (tCO_2_eq/cap) and change rates from 2010 levels (in brackets)^3)^		Emission peaking under NDC unconditional targets	
		Unconditional NDCs	Current policies	Peaking year^4)^	Average annual emissions change after peaking^5)^
Argentina	0.8%	7.1 (− 27%)	7.5 (− 23%)	2007	− 1.8%/yr (2007–2017)
Australia	1.5%	15.9 (− 41%)	19.2 (− 29%)	2007	− 1.6%/yr (2007–2019)
Brazil	3.0%	6.7 (+ 18%)	8.1 (+ 40%)	2004	− 6.5%/yr (2004–2018) (+ 1.5%/yr excl. LULUCF)
Canada	1.6%	9.5 (− 54%)	17.5 (− 20%)	2007	− 0.15%/yr (2007–2019)
China	24.6%	9.4 (+ 29%)	9.9 (+ 37%)	Before 2030 (CO_2_ only)	–-
EU-27	6.7%	4.1 (− 47%)	4.3 (− 44%)	1990 or earlier	− 1.1%/yr (1990–2019)
India	6.8%	3.0 (+ 90%)	2.3 (+ 48%)	Not expected to peak	–-
Indonesia	3.8%	6.8 (+ 45%)	6.9 (+ 47%)	Not expected to peak	–-
Japan	2.4%	6.3 (− 34%)	8.4 (− 12%)	2013	–-
Mexico	1.5%	5.5 (− 8%)	5.5 (− 8%)	Not expected to peak	–-
Republic of Korea	1.2%	8.5 (− 36%)	11.6 (− 13%)	Around 2020	–-
Russian Federation	3.7%	15.1 (+ 66%)	12.0 (+ 32%)	1990 or earlier	− 2.4%/yr (1990–2019) + 0.7%/yr (2000–2019)
Saudi Arabia	1.3%	26.3 (+ 35%)	25.0 (+ 29%)	Not expected to peak	–-
South Africa	1.1%	5.8 (− 45%)	7.2 (− 33%)	Before 2030	–-
Turkey	1.1%	10.4 (+ 131%)	5.0 (+ 11%)	Not expected to peak	–-
UK	0.9%	3.7 (− 62%)	5.4 (− 44%)	1990 or earlier	− 1.1%/yr (1990–2019)
USA	11.8%	9.3 (− 54%)	14.0 (− 30%)	2007	− 1.2%/yr (2007–2019)
G20	73.8%	6.9 (− 1%)	7.1 (+ 3%)		

^1)^GHG emissions in 2019 including emissions and removals from LULUCF based on the EDGAR database (Olivier and Peters 2020) (GHG emissions excluding those from LULUCF) and FAOSTAT (2020) (LULUCF emissions); ^2)^the population projections are based on the medium fertility variant of the UN Population Prospects 2019 edition (UN 2019); ^3)^G20 average in 2015 was 7.5 tCO_2_eq/cap. Source: this study (based on national greenhouse inventories, and using the GHG trend of EDGAR, if emission data for the most recent years were missing). ^4)^The column on peaking year of when countries are expected to peak in the future is based only on commitments that countries have made and assumes the achievement of such commitments. ^5)^Authors’ calculations based on historical emissions data (incl. LULUCF) (Section 3.2)

All countries, including those where emissions have already peaked, will need to accelerate climate change mitigation efforts if long-term reductions close to the Paris targets are to be met. For instance, an 80% reduction in emissions between 2005 and 2050 requires an annual constant reduction rate of 3.5% for that period. By contrast, the six G20 members that have already peaked have shown annualised emission reduction rates ranging between 0.15%/year (Canada) and 1.6%/year (Australia) after peaking up to 2019 for all GHG emissions including those from LULUCF. Brazil’s GHG emissions peaked in 2004 and showed an average 6.5%/year reduction between 2004 and 2018 due to the large reductions in LULUCF emissions. Brazil’s GHG emissions from non-LULUCF sectors have increased, on average, by 1.5%/year.

### Projected per capita emissions for national NDCs

Emissions per capita are a useful indicator to evaluate NDCs’ ambition. Table 2 shows projected per capita GHG emissions under current policies and NDC targets in both absolute and relative terms (compared to 2010 levels) for all G20 members (excluding EU Member States). It shows that, for six G20 members, per capita emissions are projected to exceed 10 tCO_2_eq per year (the approximate levels of 2010, for EU-27 and Japan) in 2030, under current policies and four members could even emit these levels under unconditional NDC targets. Among OECD member countries,^16^ the EU-27 and the UK are projected to have the lowest per capita emission levels in 2030 under current policies and the unconditional NDC targets, respectively. Mexico also performs well in terms of the projected development of per capita emissions under both current policies and NDC scenarios. As Table 2 shows, emissions per capita under the unconditional NDC targets are projected to decline between 2010 and 2030 in all G20 economies except China, India, Indonesia, the Russian Federation, Saudi Arabia and Turkey. There are also large differences in per capita emission levels. The per capita emissions of India and the EU-27 are about half the G20 average, whereas Saudi Arabia and the Russian Federation reach three and two times the G20 average, respectively. The per capita emissions of China reach about 35% above G20 average levels, which is above the US levels, and more than two times the level of the EU-27. Per capita emissions in G20 as a group are projected to reduce with about 5% over the 2018–2030 period in their NDCs, whereas per capita emission levels would need to be 29% and 58% lower than in 2018 to limit global warming to below 2 °C and 1.5 °C respectively. This is based on reaching global average per capita emissions of 4.9 and 2.9 tCO_2_eq by 2030 for 2 °C and 1.5 °C, respectively.^17^

### Are national NDCs in line with 2 °C and 1.5 °C pathways?

On a global level, the emission reductions that would result from implementation of the submitted NDCs are insufficient to close the global emission gap to 2 °C and 1.5 °C (see Section 4.4). On an individual country level, however, NDCs may be consistent with least-cost pathways that limit global warming to 2 °C and 1.5 °C. Consistency with emission pathways that achieve the climate targets of 2 °C and 1.5 °C, on a national level, can be assessed either by considering effort-sharing or by assuming domestic implementation of climate policies based on globally cost-optimal or least-costs scenarios.

In the first approach, emission allowances and reductions towards meeting the climate targets are distributed across countries based on alternative equity principles. Assessing whether the magnitude of change in GHG-emission-related indicators as a result of NDCs is ambitious and fair in the light of the Paris Agreement’s long-term goals requires explicit benchmarking across alternative normative indicators of effort-sharing, which was beyond the scope of this study. However, a number of recent peer-reviewed studies have attempted this task, based on a range of effort-sharing considerations (Robiou du Pont et al. 2017; van den Berg et al. 2020).

The second approach focuses on globally cost-optimal optimal scenarios for 2 °C and 1.5 °C from integrated assessment models, where emission reductions are distributed across countries, sectors and greenhouse gases in such a way that the global costs of meeting the climate targets are minimised. Note that this approach refers to the cost-optimal geographical distribution of emission reductions, not to sharing the costs of mitigation. Without such funding, the cost-optimal approach would not be seen as a fair solution. Reductions would need to be partly funded internationally, regardless of their location, to make this an equitable approach. Developed countries could reduce their emissions by more than in the cost optimal pathway and/or provide capital flows to assist poorer countries in achieving the mitigation levels shown.

**Fig. 4:**
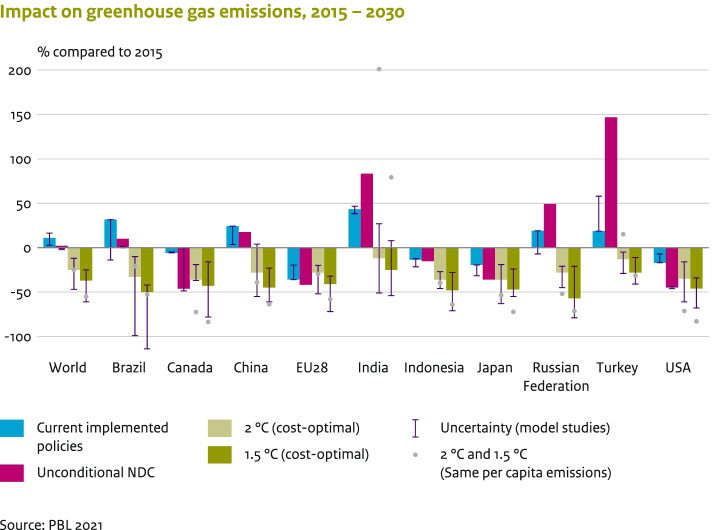
Kyoto greenhouse gas emissions by 2030 for selected regions, including the EU-27 and the UK as one region (EU28), projected by models for cost-optimal 1.5 °C and 2 °C scenarios, compared to unconditional NDCs and current policies. Total emissions are shown in comparison to 2015 levels (%, with positive numbers indicating an increase in emissions). Dots represent the emission reductions needed to meet the same world per capita emission levels for 2* °C* and 1.5 °C. Solid NDC bars show the central estimate in this study, and error bars present the range. There are three types of NDC ranges: the range for the reduction target mentioned in the NDCs themselves (‘Target’; Canada, USA), the range resulting from unconditional targets, and the range resulting from various model studies (‘Model Studies NDC’; India, China)

We compared the NDC reduction targets to projected 2030 GHG emission reductions for some major emitting countries, according to cost-optimal 2 °C and 1.5 °C scenarios developed with integrated assessment models, based on van Soest et al. (2021b), as shown in Fig. 4. Our analysis shows that only the NDCs submitted by Canada, the EU-27 and UK, and the USA are in line with a cost-optimal pathway to achieving the 1.5 °C climate target. The reduction target of the updated NDC by Japan is in line with achieving the 2 °C emission pathways. The NDCs of the other countries, including China and India, are not in line with the least-cost 2 °C pathways (let alone those of 1.5 °C). For comparison, Fig. 4 also includes the emission reductions needed to meet the same world per capita emission levels of 4.9 and 2.9 tCO_2_eq by 2030 for 2 °C and 1.5 °C, respectively, for all countries. It clearly shows the higher reductions needed for the countries with relatively high per capita emissions, such as Canada, Russian Federation and the USA, and the growth targets for India.

## Impact of changes in LULUCF on achieving NDCs

The important role of nature-based solutions for mitigating climate change is increasingly being recognised, as these may bring other important co-benefits for biodiversity and ecosystem goods and services (Seddon et al. 2020). This is partially also reflected in the updated NDCs. As many as 118 of the 143 countries assessed explicitly state that LULUCF-related emissions and their removal are included in the mitigation component of their NDCs, compared to 108 in the previous NDCs.

**Fig. 5:**
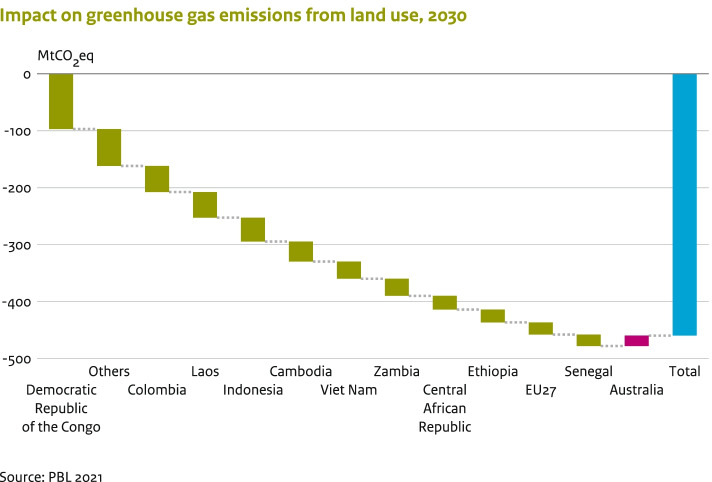
Impact of the emissions and their removal from the LULUCF sector by 2030. A negative estimate, here, reflects an enhancement of GHG commitments for the LULUCF sector within the updated NDCs, as compared to the previous NDCs. Others here shows the combined contribution for Mongolia, Papua New Guinea, Suriname, Chile, Cuba, Mexico, Macedonia, Niger, Ghana and Comoros

Overall, the updated NDC submissions amount to an additional reduction in net LULUCF emissions of 460 MtCO_2_eq by 2030, compared to the reduction commitments of the previous NDCs (Fig. 5). The additional reductions largely arise from more stringent GHG reduction targets and commitments by non-Annex I countries, with Democratic Republic of Congo in the lead with an additional emission reduction of 97 MtCO_2_eq in the LULUCF sector. Countries that provide quantitative targets in their updated NDCs represent around 20% of the world’s forest cover. However, most of the additional reductions in the LULUCF sector are not in the countries currently ranked globally in the top 10, in terms of forest area (FAOSTAT 2021).^18^ Of these top 10 countries, only Indonesia and the EU-27 have provided updated NDCs that specify additional emission reductions in the LULUCF sector, while Australia has set a lower ambition level. Brazil did not provide transparent information on its targets for the LULUCF sector, which is why its contribution could not be clearly assessed. The target of ‘realising zero illegal deforestation in Brazilian Amazonia by 2030’, as stated in Brazil’s previous NDC, is no longer included in the updated NDC. The USA is another example where the updated NDC does not provide specific targets for the LULUCF sector. As shown in Section 4, their updated NDC reduces overall emissions relative to their previous NDC target. As the USA’s updated NDC provides no information about the expected contribution from the LULUCF sector, the country is not included in Fig. 5.

The time sensitivity for mitigation actions applies particularly to the LULUCF sector. While readily available solutions are present, the promise of harnessing some LULUCF-based mitigation options may begin dissipating if no additional substantive measures are taken. As global warming increases, there is the risk of natural carbon sinks becoming sources, turning them from a solution into a problem. For example, there is evidence that, due to the combined effects of deforestation, environmental degradation and climate change, parts of the Amazon are becoming an emission source (Gatti et al. 2021), reflecting concerns that a tipping point is being reached (Nobre et al. 2016).

## Macroeconomic impacts of NDCs by 2030

Climate policies would impact the increase in economic activity in major emitting countries, as these policies affect household income, consumption of goods, trade patterns, labour markets and investment dynamics. As climate policies may have regressive distributional impacts posing a disproportionately high cost to vulnerable industries and low-income households, appropriate policies should be implemented to compensate the producers and consumers affected by decarbonisation policies, while carbon pricing itself could be used to fund these policies (Fragkos et al. 2021a). In our analysis, we did not assess compensatory schemes, as there is high uncertainty on their design and implementation, and we focused on the direct, indirect and induced socio-economic implications of implementing the new and updated NDC emission targets by 2030^19^ (all GHG emissions excluding LULUCF), by quantifying the overall economic cost of emission abatement, without considering climate damages and potential co-benefits of climate policy for air quality and human health.

Energy system restructuring induced by the new and updated NDCs leads to a replacement of fossil fuel–intensive technologies and infrastructure for low-carbon and energy-efficient technologies. Under the NDC scenario, investments in clean energy technologies increase, which puts pressure on the capital market, as capital resources are not assumed to be abundant in the CGE framework. This, in turn, will lead to ‘crowding-out effects’, as firms and households fund their clean energy investment by spending less on other, non-energy commodities and investment purposes^20^ through a reallocation of their available resources.

The economy-wide effects are driven by the uptake of low-carbon technologies and energy efficiency, leading to increases in the cost of energy services, under the current policies (CurPol) scenario of the GEM-E3-FIT macroeconomic model. This induces an increase in the price of goods and services and a decline in the purchasing power of households, thus, reducing domestic demand. Full implementation of the NDC targets implies substituting fossil fuels (which are largely imported into the EU-27, Japan, China and India) with capital-intensive goods and services that are, to a certain extent, domestically produced. Spending on low-carbon technologies constrains the funds available for other investments and consumption purposes (crowding-out effect). The model-based analysis suggests that the new and updated NDCs will have only a limited impact on economic activity, with global GDP declining by 0.4% from CurPol levels, by 2030 (Table 3). This is in line with earlier macroeconomic assessments of the previous NDC submissions (i.e. Vandyck et al. (2016); Fragkos et al. (2018)), despite achieving larger emission reductions, as GEM-E3-FIT has integrated the declining costs of renewable energy technologies, in recent years. The economic costs that will emerge from the NDCs can be lower, if the benefits related to avoided climate impacts and air pollution are explicitly quantified (Rauner et al. 2020).

**Table 3:** Macroeconomic impacts of the new NDCs in major economies (as % changes from the CurPol scenario by 2030). Source: GEM-E3-FIT model

	GDP	Investment	Consumption	Employment	% GHG emission reduction* from CurPol in 2030
EU-27	− 0.5	0.3	− 0.5	− 0.5	− 15%
USA	− 0.8	− 0.7	− 1.8	− 0.8	− 29%
China	− 0.5	− 0.5	− 1.1	− 0.5	− 7%
India	0.0	0.1	0.1	0.0	0%
Japan	− 0.6	− 0.5	− 1.2	− 0.7	− 23%
Russian Federation	− 0.1	− 0.1	− 0.1	0.1	0%
Canada	− 0.6	− 0.5	− 0.9	− 0.6	− 25%
South Africa	0.1	0.2	0.2	0.2	0%
Republic of Korea	− 0.5	− 0.2	− 0.9	− 0.4	− 21%
Mexico	− 0.5	− 0.9	− 2.0	− 0.4	− 18%
Argentina	− 0.1	0.1	0.0	0.0	− 3%
Turkey	0.1	0.1	0.2	0.1	0%
Saudi Arabia	− 0.6	0.0	− 0.3	− 0.2	0%
Australia	− 0.6	− 0.4	− 1.0	− 0.5	− 15%
World	− 0.4	− 0.2	− 0.7	− 0.2	− 12%

^*^All GHG emissions excluding LULUCF

Macroeconomic impacts of NDCs differ between countries, largely depending on the emission reduction effort relative to CurPol, which is in line with the analysis presented above. Other country-level factors also influence mitigation costs, such as economic structure, fossil fuel resources, energy transformation, position in global energy trade, and technology costs. Overall, fossil fuel exporters face higher costs relative to importers, as a result of their reduced export revenues, while fossil fuel import savings reduce the mitigation costs for energy importers. GDP losses are found to be larger in the USA (0.8% of CurPol GDP in 2030) due to the higher ambition level of the new US NDC emission reduction target of 50–52%. GDP impacts are marginal for countries whose NDCs do not lead to emission reductions from CurPol — excluding LULUCF emissions (e.g. India, Russian Federation, Argentina, Turkey, South Africa). The new, more ambitious NDC targets lead to limited GDP losses of around 0.5%, by 2030, in major developed economies, including the EU-27, Japan, Canada, the Republic of Korea and Australia, while China faces similar losses, mostly driven by the Chinese updated NDC (Table 1), as well as by the reduced exports to other economies due to lower global output, given that China plays a large role in the global trade of industrial commodities. Overall, increases in investment costs of low-carbon technologies and infrastructure are to a large extent offset by fossil fuel savings, with a transition towards a more capital intensive economy. Our analysis shows that the new NDC targets are compatible with robust economic growth in all G20 economies, as GDP growth rates are found to remain very close to CurPol levels, with only marginal changes by 2030.^21^

The increased low-carbon investment, under the NDC scenario, crowds out investment in other productive activities, as discussed above, leading to a small reduction in global investment, which declines by 0.2% from CurPol by 2030, driven by output reductions. The NDC impacts on investment are more limited than those on GDP (in all countries), due to increased investments in low-carbon technologies and energy efficiency, in line with Fragkos et al. (2021b) and European Commission (2020), implying a transition to a more capital-intensive structure of the economy. The net impact on investment is determined by the interplay of output reductions and the higher capital intensity of decarbonisation. In the case of EU, GDP impacts are relatively lower than in most major economies (USA, China, Japan) while the additional energy system investments required to achieve the ambitious NDC target of 55% emission reductions by 2030 are very high, leading to increased aggregate investments. In all countries, the impact of the implementation of NDCs on investment is relatively more positive than the impact on GDP and consumption.

On the other hand, private consumption decreases more than GDP (0.7% globally, between 0.1 and 1.2% in major economies), as production costs and prices increase due to carbon pricing, energy system restructuring and resource reallocation, compared to those under the CurPol scenario. The overall impact of the new NDCs on employment is limited, with global employment declining by 0.2% from the level under the CurPol scenario by 2030, with deviations between countries ranging from − 0.8 to + 0.1%, which largely reflect the mitigation ambition level of the new NDCs. The NDC impacts on employment are projected to be more limited than those on GDP, as job impacts from reduced economic activity are largely counterbalanced by the creation of jobs in sectors related to low-carbon technologies and energy efficiency, as these sectors are commonly more labour-intensive than the fossil fuel sector (Fragkos and Paroussos 2018).

## Conclusions

This paper analyses the emissions and macroeconomic impacts of the new and updated NDCs submitted between October 2020 and January 2022, where available and previous NDCs for others, within the context of the Paris Agreement. The following main conclusions can be drawn from the analysis.

The NDC updates — unconditional and conditional — have an aggregated impact on global GHG emissions of about − 3.8 and − 3.9 GtCO_2_eq by 2030 respectively, compared to the previous NDCs. Nine G20 economies (USA, China, the EU-27, Japan, South Africa, the UK, Argentina, Canada and the Republic of Korea) have pledged lower emission targets for 2030, in their updated NDCs, leading to additional aggregated GHG emission reductions of about 3.2 GtCO_2_eq, compared to the previous NDCs. About 0.3 GtCO_2_eq will be counterbalanced by the higher emissions from a group of countries with a change in base year or BAU emissions, the most prominent of which being Brazil. The non-G20 economies with updated NDCs contribute a reduction of 0.8 GtCO_2_eq.

The collective ambition level of all submitted NDCs (also including the updates) falls short of what is needed to put global emissions on to a cost-effective pathway towards achieving the climate goals of the Paris Agreement. Global emissions are projected to peak by 2025 and reach about 52.3 (50.1–54.1) GtCO_2_eq, by 2030, if all unconditional NDCs are implemented, and 50.7 (48.5–52.2) GtCO_2_eq if the conditional NDCs are implemented. The median estimates of unconditional NDCs for 2030 are 2% above 2015 emission levels. By 2030, global emission levels need to be 10.8 (4.1–15.4) GtCO_2_eq lower than current unconditional NDCs imply, in order to achieve the 2 °C goal, and 27.6 (19.2–31.8) GtCO_2_eq lower for the 1.5 °C goal. Implementation of the conditional NDCs would reduce these estimates by about 1.5 GtCO_2_eq. While the NDCs are projected to be insufficient for closing the global emission gap, the NDCs of some G20 economies (i.e. Canada, the European Union and USA) are consistent with emission pathways below 2 °C and 1.5 °C, based on cost-optimal implementation.

Four G20 members are projected to emit more than 10 tCO_2_eq per capita, annually (2010 levels for EU-27 and Japan) by 2030, under unconditional NDCs. Emissions per capita, under the unconditional NDCs, are projected to decline between 2010 and 2030 in all G20 economies except China, India, Indonesia, the Russian Federation, Saudi Arabia and Turkey. There are also large differences in per capita emission levels. The per capita emissions in India and the EU-27 are about half the G20 average, whereas Saudi Arabia and the Russian Federation reach three and two times the G20 average, respectively.

The new NDCs will have a limited socio-economic impact in G20 economies. The new, updated NDCs will have only limited impact on economic output in major economies with GDP losses crucially depending on the level of emission reduction effort by 2030. The economic impact highly depends on the ambition level of NDC targets and on a country’s trade position on global energy and other goods markets. The new NDCs will lead to a decline in global GDP of 0.4% compared to the current policies scenario, while GDP losses in major economies by 2030 range between 0.0% (when NDCs do not imply climate efforts in addition to those under the current policies scenario) and 0.8% (in the USA, with its high mitigation effort and fossil fuel production).

The updated NDCs show a broader scope of mitigation efforts, with most countries providing cross-sectoral targets and a greater inclusion of the LULUCF sector. The updated NDC submissions show an increase in both mitigation scope and ambition level of the LULUCF-related measures. However, when taken together with mitigation actions across other sectors, the size and pace of nationally determined emission reductions currently will not meet the collective commitments required to achieve a pathway that aligns with the objectives of the Paris Agreement. This underlines the urgency of scaling up further actions, if a global warming limit of between 1.5 and 2 °C is to remain within the realm of possibilities. Further transparency and clear description of how much the LULUCF sector will contribute to the overall emission reduction target will help in recognising changes in scope and identifying entry points for action, particularly from key forest countries.

A continuous shortfall in collective ambition may foreclose some existing mitigation options, placing an additional burden on the future. Based on our assessment, the impact of the updated NDCs would need to be three times greater to be consistent with temperature increases of well below 2 °C, and about seven times greater for 1.5 °C. The urgency for scaling up the ambition level needs to be recognised in the context of the time sensitivity and, hence, also the need for prioritisation of mitigation options. With further warming and increases in weather extremes, some of the mitigation options readily available today may no longer be viable at a later point in time. This may apply to forest sinks or the use of hydropower, in some regions. Hence, shortfalls in climate ambition levels today may translate into an additional demand for novel technologies and practices in the future, placing a further burden and challenges on future generations.

### Electronic supplementary material

Below is the link to the electronic supplementary material.

Supplementary file1 (DOCX 395 KB)

## Data Availability

An interactive tool showing emissions per country, emissions per capita, and emissions per unit of income, resulting from the current policies and NDCs, is available at www.pbl.nl/ndc. All data of figures used in the analysis will be made available in a data repository upon publication.

## Appendix Modelling framework

### A.1 The IMAGE integrated assessment model

The IMAGE model is an integrated assessment model with 26 regions as well, consisting of a set of linked and integrated models, which, together, describe important elements in the long-term dynamics of global environmental change (Hof et al. 2022; Stehfest et al. 2014; van Vuuren et al. 2017a, 2018). The model includes detailed descriptions of the energy system and food consumption and production, using TIMER and agricultural trade and production models. The land-cover submodels simulate the change in land use and land cover, at 0.5 × 0.5° (driven by demand for food, timber and bioenergy, and changes in climate), and the land-use-related emissions. In terms of land-based mitigation options, IMAGE accounts for three general types of options: bio-energy production, REDD (avoided deforestation) and reforestation of degraded forests. Bio-energy demand is determined by TIMER based on bio-energy yield, the carbon price, dynamics in the energy system, and land availability, following a food-first principle.

### A.2 The TIMER energy model

The *TIMER* energy model of IMAGE has been developed to explore scenarios for the energy system in terms of trends in energy demand and efficiency and the possible transition towards renewable energy sources (van Vuuren 2007; van Vuuren et al. 2017a). TIMER describes 12 primary energy carriers in 26 world regions and analyses long-term trends in energy demand and supply. It covers a wide range of mitigation options, including nuclear power, renewable energy (different solar and wind technologies, hydropower), bioenergy (first and second-generation), nuclear power and CCS technology (van Vuuren et al. 2017a). The model’s behaviour is mainly determined by substitution processes of various technologies based on long-term prices and fuel preferences. These two factors drive multinomial logit models that describe investments in new energy production and consumption capacity. The long-term prices that drive the model are determined by resource depletion and technological development. Resource depletion is important for fossil fuels (for which depletion and costs depend on annual production rates). Bio-energy is available as a substitute of fossil fuels for both liquid fuel and thermal generation. Technological development is determined by learning curves or through exogenous assumptions. Emissions from the energy system are calculated by multiplying energy consumption and production flows with emission factors. A carbon tax can be used to induce a dynamic response such as increased use of low or zero-carbon technologies, energy efficiency improvement and end-of-pipe emission reduction technologies.

### A.3 The GLOBIOM and G4M land-use models

The Global Biosphere Management Model (*GLOBIOM*) is a partial equilibrium model of the global agricultural and forestry sectors (Havlík et al. 2014) that is applied in combination with the Global Forest Model (*G4M*) (Fricko et al. 2017; Kindermann et al. 2008). GLOBIOM models commodity markets and international trade at a regional level, which establishes market equilibrium by endogenous price determination at the regional level. On the supply side, the model relies on simulation units, which comprise aggregates of 5–30 arcmin pixels whose slope, altitude and soil class are the same, as is the same country. The model is solved in a recursive dynamic manner for time steps of 10 years, which results in decade-wise detailed trajectories for variables related to supply, demand, prices, land use and AFOLU emissions. CO_2_ emissions and removals from afforestation, deforestation and wood production in managed forests are estimated by the geographically explicit (0.5 × 0.5°) G4M model which is connected with GLOBIOM. In G4M, afforestation and deforestation decisions are based on a comparison of agricultural and forestry land value net present values. Afforestation occurs where it is more profitable than the agriculture production and the environment supports the establishment of forests. Contrary to this, deforestation occurs when net present values from agriculture plus profits from one-time sales of deforested wood exceed net present values from forestry.

### A.4 The GEM-E3-FIT macro-economic model

The *GEM-E3-FIT* model is a multi-regional, multi-sectoral, recursive dynamic computable general equilibrium (CGE) model which provides details on the macro-economy and its interaction with the environment and the energy system. It is an empirical, large-scale model, written entirely in structural form. GEM-E3 allows for a consistent comparative analysis of policy scenarios since it ensures that in all scenarios, the economic system remains in general equilibrium. In addition, it incorporates micro-economic mechanisms and institutional features within a consistent macro-economic framework. Particularly valuable are the insights the model provides regarding the distributional aspects of long-term structural adjustments. The GEM-E3-FIT model is extensively used as a tool of policy analysis and impact assessment (Paroussos et al. 2020). The model is dynamic, recursive over time, driven by accumulation of capital and equipment. The economic agents are assumed to exhibit optimising behaviour while market-derived prices are adjusted to clear all markets. The model features alternative market regimes, discrete representation of power producing technologies, equilibrium unemployment, energy efficiency standards and carbon pricing. GEM-E3-FIT formulates production technologies in an endogenous manner allowing for price-driven derivation of intermediate consumption and the services from capital and labour. The model can quantify the macroeconomic, trade, employment and distributional impacts of environmental and energy policies. For alternative scenarios, GEM-E3-FIT provides for 51 countries (including G-20 economies): dynamic projections of national accounts; full input–output tables; distribution of income and transfers by agent; employment by economic activity and by skill; investment by sector; CO_2_ and GHG emissions by sector and fuel; full bilateral trade matrices among countries and sectors; energy demand and supply by sector and fuel, energy efficiency investment and power generation mix.

## Footnotes

1 In the analysis, we consider the European Union and its three individual Member States (France, Germany and Italy) as one member.

2 Including 95 countries with updated NDCs, and for the other 48 countries previous NDCs were used.

3 An interactive tool showing emissions per country, emissions per capita, and emissions per unit of income, resulting from the current policies and NDCs, is available at www.pbl.nl/ndc.

4 All GHG emission figures are expressed using the 100-year global warming potential (GWPs) from the IPCC Fourth Assessment Report.

5 www.climatepolicydatabase.org

6 For countries that are part of a larger IMAGE region (Australia, Kazakhstan, the Republic of Korea, Russian Federation and Ukraine), emission projections were scaled down, using the country’s share in the region’s 2015 emissions as a constant scaling factor.

7 Chile, Colombia, Egypt, Ethiopia, Iran, Kazakhstan, Morocco, the Philippines, Thailand, Ukraine, United Arab Emirates, Vietnam.

8 The unconditional target holds irrespective of actions of other countries, whereas for the latter, more ambitious, target, certain conditions regarding the actions of other countries (which mostly refer to international finance or international cooperation mechanisms) have to be met (den Elzen et al. 2016; UNFCCC 2022).

9 Chile, Colombia, Egypt, Ethiopia, Iran, Kazakhstan, Morocco, the Philippines, Thailand, Ukraine, United Arab Emirates, Vietnam.

10 Both models have implemented the same modelling protocol for current policies (Roelfsema et al. 2022), updated from Roelfsema et al. (2020), including a detailed spreadsheet listing policies by country to implement current policies, leading to (limited) differences in the emissions projections (Roelfsema et al. 2020).

11 The uncertainty range comes from the different current policies projections in Nascimento et al. (2021).

12 The Fourth BUR of Brazil gives 2005 emissions of 2,7 GtCO_2_eq expressed in GWPs from the IPCC SAR, as can be seen on page 45 (BUR). For this analysis, this estimate is corrected for GWPs from the IPCC AR4, leading a base-year emissions of 2.8 GtCO_2_eq, which is also confirmed in the BUR.

13 This is also found in the model assessment of independent national and global studies by den Elzen et al. (2019).

14 Meinshausen et al. (2022) have also analysed the projected GHG emissions (excluding LULUCF) resulting from the new or updated NDCs. They found projected global GHG emissions of 7 to 15% above 2010 levels, which are close to the projections of this study.

15 Russia’s emissions peaked prior to 1990. While Russia’s commitments for 2020 and 2030 indicate an intended increase from recent emissions levels, Russia’s future commitments do not propose to surpass 1990 emissions levels.

16 Australia, Canada, the EU, Japan, Mexico, the Republic of Korea, Turkey, the UK and the USA.

17 By 2030, global GHG emissions would need to be 25% and 55% lower than in 2018 to put the world on the least-cost pathway to limiting global warming to below 2 °C and 1.5 °C respectively.

18 Russian Federation, Brazil, Canada, the Unites States, China, EU-27, Australia, Democratic Republic of the Congo and Indonesia.

19 The updated NDC of South Africa (submitted by 27 September 2021) was not included in the macro-economic analysis.

20 Crowding-out effects can diminish in cases of favourable financing schemes, as assumed in Fragkos and Paroussos (2018), who show that, if firms and households can borrow on capital markets without facing an increase in the related costs, the activity impacts of decarbonisation will be minimal or even positive (in the short term). In addition, neo-Keynesian assumptions do not include constraints in the financial capacity of agents and, thus, show no crowding-out effects as a result of decarbonisation policies, at least not in the short term.

21 The global GDP annual growth rate marginally declines from 2.6% in CurPol to 2.5% in the NDC scenario over 2015–2030.
