# Supplementary material for: Updated nationally determined contributions collectively raise ambition levels but need strengthening further to keep Paris goals within reach
Source: Mitig Adapt Strateg Glob Chang. 2022 Jun 21;27(5):33. doi: 10.1007/s11027-022-10008-7 (PMC9209833; doi:10.1007/s11027-022-10008-7)
Supplement: Supplementary file 1 — Supplementary file1 (DOCX 395 KB) [file 11027_2022_10008_MOESM1_ESM.docx]

Supporting Information

Updated nationally determined contributions collectively raise ambition levels but need strengthening further to keep Paris goals within reach

Contents

[Supplementary Text 1: Country classification according to type of target 2](#_Toc84076482)

[Supplementary Text 2: Overview of the mitigation contribution of submitted NDCs 4](#_Toc84076483)

[Supplementary Text 3: Projected greenhouse gas emission targets levels from the NDCs of the G20 economies by 2030 12](#_Toc84076484)

[Supplementary Text 4: LULUCF accounting method chosen for quantification 14](#_Toc84076485)

[Supplementary Text 5: Methodology for the current policies scenario 10](#_Toc84076486)

[Supplementary Text 6: Projected global greenhouse gas emission levels by 2030 16](#_Toc84076487)

[Supplementary Text 7: GHG reduction targets for the G20 economies of the NDC including and excluding the LULUCF sector 18](#_Toc84076488)

[Supplementary Figure 1: The contribution of the updated NDC targets of countries on total additional global emissions reduction 19](#_Toc84076489)

[References 20](#_Toc84076490)

# Supplementary Text 1: Country classification according to type of target

In advance of the Paris meeting, almost all countries submitted their national post-2020 climae action plans and commitments, as part of their Intended Nationally Determined Contributions (INDCs). Over about 195 countries have ratified the agreement; thus, turning their INDCs into NDCs. The following countries have not ratified the Paris Agreement, i.e.: Eritrea, Iran, Iraq, Libya and Yemen. The mitigation components of the NDCs represent several types of targets (Figure S.1), as summarised below ([ClimateWatch, 2022](#_ENREF_2); [UNFCCC, 2022](#_ENREF_17)):

1. **Base year target: economy-wide absolute reduction from historical base year emissions.** NDCs report on an absolute reduction from historical base year emissions. The base year chosen varies, with 1990, 2005 and 2010 being the most common.
2. **Baseline or BAU target: emission reductions relative to a baseline or business-as-usual projection (specified in the NDCs).** The type of emission reduction relative to a baseline or business-as-usual projection has been chosen for many NDCs. The mitigation component of the NDCs specifies the business-as-usual emission projection.
3. **Baseline or BAU target (not specified): emission reductions relative to a baseline projection (not specified).** Same as under point 2, but here, for the NDCs, baseline or business-as-usual emission projections are not specified, such as for those of the Philippines and Venezuela. For the calculations, we used the baseline projections from national studies (when available) and the PBL business-as-usual projections.
4. **Intensity target.** At least five countries, including Malaysia, in their NDCs, indicate reductions in emission intensity in GDP as the main type of mitigation.
5. **Intensity and non-GHG target: emission intensity target and non-greenhouse gas target.** China and India aim for emission intensity improvements, a target for non-fossil fuels in primary energy consumption/power capacity, and for China, a target year for the peaking of emissions.
6. **Trajectory and fixed-level target.** South Africa has a trajectory target stating the emission ranges for 2025 and 2030. Several countries, such as Israel and Ethiopia, put forward a fixed-level target, specifying the MtCO_2_eq that they intend not to exceed in a given year.
7. **Submitted actions (cannot be quantified).** Finally, many countries include mere qualitative descriptions of mitigation actions in their NDCs, or specific targets for sub-sectors, such as for the implementation of renewable energy. As such targets complicate a precise quantification, we have not analysed them here. This group of countries covers about 5% of the global emissions of 2019.


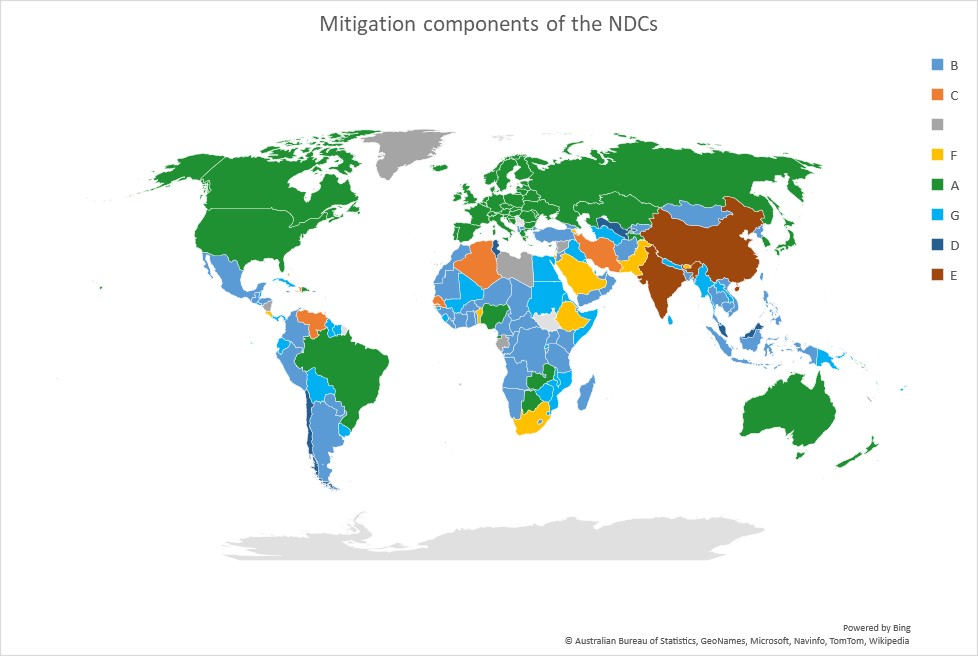


Figure S.1. – Map showing the mitigation components of the NDCs representing several types of target (see main text).

# Supplementary Text 2: Overview of the mitigation contribution of submitted NDCs

Table S.1 presents an overview of the mitigation targets of most NDCs submitted ([ClimateWatch, 2022](#_ENREF_2); [UNFCCC, 2022](#_ENREF_17)). As on 31 January 2022, about 155 countries have submitted new NDC targets (see Table S.1). For the analysis, we assessed the mitigation components of the NDCs of 143 countries, including the EU-27 as a group (representing approximately 96% of global greenhouse gas emissions in 2019, excluding international aviation and shipping emissions). Other countries are not included in this analysis because of too much uncertainty in the quantification of their NDCs, or because their 2018 emission share was less than 0.1%.

Table S.1. Overview of the mitigation targets of the NDCs submitted by 15 December 2021, including the share of each party in 2018 global greenhouse emissions (parties listed in alphabetical order). The Table also shows the NDCs that are included in the analysis (column three), with labels (A-G) explained above.

| Country | Share GHG emissions 2019^1)^ | Cate-gory ^2)^ | Updated NDC? (++: reduced emissions) ^2)^ | GHG emission reduction target^3)^ | | |
| --- | --- | --- | --- | --- | --- | --- |
| Afghanistan | 0.07% | B |  | Conditional: 13.6% | By 2030 | Below BAU |
| Albania | 0.02% | C | Yes (++) | 20.9% GHG emissions | By 2030 | Below BAU |
| Algeria | 0.50% | C |  | Conditional: 7% to 22% | By 2030 | Below BAU |
| Andorra | <0.01% | B | Yes | 37% | By 2030 | Below BAU |
| Angola | 0.29% | B | Yes (++) | -14% (conditional: -24%) | By 2030 | Below BAU |
| Antigua and Barbuda | <0.01% | G |  | List of policies and measures | By 2030 | Below 2006 |
| Argentina | 0.76% | F | Yes (++) | An economy-wide unconditional cap net emissions of 359 MtCO_2_eq in 2030. Net emissions cap of 349 MtCO_2_eq in 2030 | By 2030 | Fixed target |
| Armenia | 0.02% | A | Yes (++) | 40% | By 2030 | Below 1990 |
| Australia | 1.5% | A | Yes | 26% to 28% | By 2030 | Below 2005 |
| Azerbaijan | 0.11% | A |  | 35% | By 2030 | Below 1990 |
| Bahamas | 0.01% | C |  | Conditional: 30% | By 2030 | Below BAU |
| Bahrain | 0.08% | G | Yes | List of policies and measures | - | - |
| Bangladesh | 0.52% | B | Yes (++) | 7% (conditional: 15%) | By 2030 | Below BAU |
| Barbados | <0.01% | B | Yes (++) | 35% (70% by 2030) | By 2030 | Below 2008 |
| Belarus | 0.24% | A | Yes (++) | 35% (conditional: 40%) | By 2030 | Below 1990 |
| Belize | <0.01% | G | Yes | Conditional: -24 MtCO_2_eq | 2014 - 2033 | below BAU |
| Benin | 0.06% | C | Yes | Conditional: 21.4% | By 2030 | Below BAU |
| Bhutan | 0.01% | G | Yes | Intends to remain carbon neutral | - | Fixed Level Target |
| Bolivia | 0.25% | G |  | No explicit target for emission reduction | - | - |
| Bosnia-Herzegovina | 0.07% | A | Yes (++) | 13% (conditional: 17.5%) | by 2030 | below 1990 |
| Botswana | 0.27% | A |  | 15% | By 2030 | Below 2010 |
| Brazil | 3.02% | A | Yes | 37% below 2005 in 2025 (43% below 2005 levels in 2030) | By 2025 (By 2030) | Below 2005 |
| Brunei | 0.03% | G | Yes | Land Transport sector: to reduce CO_2_ emissions from morning peak hour vehicle use by 40% | By 2030 | Below BAU |
| Burkina Faso | 0.10% | B | Yes (++) | 19.6% (conditional: 29.4%) | By 2030 | Below BAU |
| Burundi | 0.02% | E | Yes (++) | 3% (conditional 13%) | By 2030 | Below BAU |
| Cape Verde | <0.01% | G | Yes | 30% renewables, 10% energy savings (conditional 100% renewables, 20% energy savings) | By 2025 | Increasing the share of renewable energy and energy savings |
| Cambodia | 0.15% | B | Yes | Conditional 42% reduction below BAU by 2030; a LULUCF contribution: 50% reduction below BAU by 2030 | By 2030 | Below BAU |
| Cameroon | 0.16% | B | Yes (++) | Conditional: 35% | By 2030 | Below 2010 |
| Canada | 1.58% | A | Yes (++) | 40-45% | By 2030 | Below 2005 |
| Central African Republic | 0.09% | B | Yes (++) | 11.8% (Conditional 24.3%) | By 2030 | Below BAU |
| Chad | 0.20% | B |  | 20.5% (conditional: 30%) | By 2030 | Below BAU |
| Chile | 0.12% | F | Yes (++) | a target of 95 MtCO_2_eq in 2030; peak GHG emissions by 2025 | By 2030 | Fixed target |
| China | 24.6% | E | Yes (++) | CO_2_ peaking before 2030; 65% CO_2_ intensity improvements, 25% non-fossil fuels in primary energy consumption, increase the forest stock volume; list of policies and measures. | By 2030 | Below 2005 |
| Colombia | 0.50% | B | Yes (++) | Conditional: 51% | By 2030 | Below BAU |
| Comoros | <0.01% | B | Yes (++) | reduce GHG emissions excluding LULUCF by 23% and increase CO_2_ removals by 47% | By 2030 | Below BAU |
| Cook Islands | <0.01% | G |  | 38% (conditional: 81%) in the energy sector | By 2020 (2030) | Below 2006 |
| Congo | 0.05% | B | Yes (++) | Conditional: at least 48% (55%) | By 2025 (By 2035) | Below BAU |
| Costa Rica | 0.01% | A | Yes (++) | Net emissions 9.374 Mt CO_2_-e | by 2030 | Fixed Level Target |
| Côte d’Ivoire | 0.11% | B |  | 28% | By 2030 | Below BAU |
| Cuba | 0.09% | G | Yes | List of policies and measures | - | - |
| DR Congo | 1.26% | B | Yes | 2% (conditional: 21%) | By 2030 | Below BAU |
| Djibouti | 0.01% | C |  | 40% (conditional: 60%) | By 2030 | Below BAU |
| Dominica | <0.01% | A |  | Conditional: 17.9% by 2020; 39.2% by 2025 & 44.7% by 2030 | By 2030 | Below 2014 |
| Dominican Republic | 0.07% | A | Yes | 7% (conditional: 27%) | By 2030 | Below 2010 |
| Ecuador | 0.18% | G |  | 20.4% to 25% (conditional: 37.5% to 45.8%) | by 2025 | Below BAU |
| Egypt | 0.65% | G |  | List of policies and measures | By 2030 | - |
| El Salvador | 0.02% | G | Yes | Commits to reducing energy sector emissions by between 640 ktCO_2_eq (unconditional) and 819 ktCO_2_eq (conditional) | By 2030 | Below BAU |
| Equatorial Guinea | 0.02% | A |  | 20% (with a view to 50% by 2050). Conditional on (unspecified) technical & financial support | By 2030 (By 2050) | Below 2010 |
| Eritrea | 0.01% | G |  | 39.2% (conditional: 80.6%) | By 2030 | Below BAU |
| Ethiopia | 0.38% | B | Yes (++) | 14% (conditional: 69%) | By 2030 | Below BAU |
| EU28 | 7.58% | A | Yes (++) | At least 55% domestic GHG emission reduction from 1990 | By 2030 | Below 1990 |
| Fiji | <0.01% | G | Yes (++) | List of policies and measures | By 2030 | Below BAU |
| Macedonia | 0.02% | C | Yes (++) | 51% compared to 1990 levels, or 82% if expressed in net emissions | By 2030 | Below 1990 |
| Gabon | 0.04% | C |  | 50% | By 2025 | Below BAU |
| Gambia | 0.01% | C | Yes | Conditional actions only | 2021 - 2025 | N.A. |
| Georgia | 0.04% | B | Yes (++) | 35% (conditional: 50-57%) | By 2030 | Below 1990 |
| Ghana | 0.03% | B | Yes | 15% (conditional: 45%) | By 2030 | Below BAU |
| Grenada | <0.01% | A | Yes (++) | 30% (indicative: 40% below 2010 levels by 2030) | By 2025 (By 2030) | Below 2010 |
| Guatemala | 0.08% | B |  | 11.2% to 22.6% | By 2030 | Below BAU |
| Guinea | 0.07% | G | Yes (++) | 10% (conditional: 17%) | By 2030 | Below BAU |
| Guinea Bissau | 0.01% | G | Yes | No explicit target for emission reduction | - | - |
| Guyana | 0.03% | G |  | Up to 52Mt CO_2_ (20% renewables) | By 2025 | Below BAU |
| Haiti | 0.03% | C |  | 5% (conditional: 26%) | By 2030 | Below BAU |
| Honduras | 0.05% | B | Yes (++) | Conditional: 16% | By 2030 | Below BAU |
| Iceland | 0.01% | A | Yes (++) | at least 55% | By 2030 | Below 1990 |
| India | 6.81% | E |  | Conditional: 33% to 35% emission intensity improvement; renewable energy to increase to 40% of total power capacity and an additional carbon sink of 2.5 to 3 Mt CO_2_eq through additional forest and tree cover | By 2030 | Below 2005 |
| Indonesia | 3.76% | B | Yes | 29% (conditional: 41%) | By 2030 | Below BAU |
| Iran | 1.70% | C |  | 4% (conditional: 12%) | By 2030 | Below BAU |
| Iraq | 0.55% | B | Yes | 1% (conditional: 13%) | By 2030 | Below BAU |
| Israel | 0.16% | A | Yes (++) | 27% | By 2030 | Below 2015 |
| Jamaica | 0.02% | B | Yes (++) | 7.8% (conditional: 10%) | By 2030 | Below BAU |
| Japan | 2.45% | A | Yes (++) | 46% | By 2030 | Below Fiscal year 2013 |
| Jordan | 0.07% | B | Yes (++) | 5% (conditional: 26%) | By 2030 | Below BAU |
| Kazakhstan | 0.7% | A |  | 15% (conditional: 25%) | By 2030 | Below 1990 |
| Kenya | 0.16% | B | Yes (++) | Conditional: 32% | By 2030 | Below BAU |
| Kiribati | <0.01% | B |  | 12.8% (13.7% by 2025) | By 2030 (by 2025) | Below BAU |
| Kuwait | 0.25% | G | Yes | List of policies and measures | By 2035 | - |
| Kyrgyzstan | 0.04% | C | Yes (++) | 15.97% (conditional: 43.6%) (also includes 2050 goals) | By 2030 | Below BAU |
| Laos | 0.06% | G | Yes | 60% (conditional targets set for land use, energy, agriculture, and waste sectors) | By 2030 | Below BAU |
| Lebanon | 0.06% | B | Yes (++) | 20% (conditional 31%) | By 2030 | Below BAU |
| Lesotho | 0.01% | C |  | 10% (conditional 35%) | By 2030 | Below BAU |
| Liberia | 0.03% | B | Yes | 10% (conditional 64%) | By 2030 | Below BAU |
| Liechtenstein | <0.01% | A |  | 40% | By 2030 | Below 1990 |
| Madagascar | 0.08% | B |  | Conditional: 14% | By 2030 | Below BAU |
| Malawi | 0.04% | G | Yes | No explicit target for emission reduction | - | - |
| Malaysia | 0.75% | D | Yes (++) | 45% emission intensity improvement | By 2030 | Below 2005 |
| Maldives | <0.01% | B | Yes | conditional: 26% | By 2030 | Below BAU |
| Mali | 0.08% | G | Yes (++) | reduce emissions by 31% for energy, 25% for agriculture, 39% for land use and forestry, and 31% for waste sectors | By 2030 | Below BAU |
| Marshall Islands | <0.01% | A | Yes | 45% below 2010 levels by 2030 | By 2030 | Below 2010 |
| Mauritania | 0.02% | B |  | 22.3% (33.6 MtCO_2_eq) of which 88% conditional | By 2030 | Below BAU |
| Mauritius | 0.01% | B | Yes (++) | Conditional: 40% | By 2030 | Below BAU |
| Mexico | 1.49% | B | Yes | 22% (conditional: 36%), emissions peaking after 2026 | By 2030 | Below BAU |
| Micronesia | <0.01% | A |  | 28% (conditional: 35%) | By 2025 | Below 2000 |
| Monaco | 0.03% | A | Yes | 55% | By 2020 | Below 1990 |
| Mongolia | 0.15% | B | Yes | 23% (Conditional: 27%) | by 2030 | below BAU |
| Montenegro | <0.01% | A | Yes (++) | 35% | by 2030 | below 1990 |
| Morocco | 0.18% | B | Yes (++) | 18.3% (conditional: 45.5%) | By 2030 | Below BAU |
| Mozambique | 0.22% | G | Yes | 22.7% (conditional: 27.2%) | By 2030 | Below BAU |
| Myanmar | 0.50% | G | Yes | reduce cumulative GHG emissions over 2021-2030 by 244.52 MtCO_2_eq (unconditional) and 414.75 MtCO_2_eq (conditional) | By 2030 | Below BAU |
| Namibia | 0.05% | B | Yes | 14% (Conditional: 91%) | By 2030 | Below BAU |
| Nauru | <0.01% | G |  | List of policies and measures | By 2030 | - |
| New Zealand | 0.14% | A | Yes (++) | 50% below gross 2005 levels | By 2030 | Below 2005 |
| Nepal | 0.11% | G | Yes | commits targets and measures in energy, industry, agriculture, forestry and land use, and waste sectors | - | - |
| Niger | 0.07% | B | Yes | Reduce emissions in the AFOLU sector by 12.57% (unconditional), and by 22.75% (conditional). Reduce emissions in the energy sector by 10.6% by 2030 (unconditional), and 45% by 2030 (conditional). | By 2030 | Below BAU |
| Nigeria | 0.67% | B | Yes (++) | 20% (conditional: 47%) | By 2030 | Below BAU |
| Niue | <0.01% | G |  | List of actions: 38% Renewables  Conditional: at least 80% | By 2020  By 2025 | - |
| North Korea | 0.09% | B | Yes | 16.4% (Conditional: 52%) | By 2030 | Below BAU |
| Norway | 0.10% | A | Yes (++) | Reduce emissions by at least 50% by 2030, aiming towards 55% below 1990 levels | By 2030 | Below 1990 |
| Oman | 0.22% | B | Yes | 4% (Conditional: 7%) | By 2030 | Below BAU |
| Pakistan | 0.91% | B |  | 15% (Conditional: 35%) | By 2030 | Below BAU |
| Palau | <0.01% | G |  | 22% energy sector emissions reductions; energy target | By 2025 | Below 2005 |
| Panama | 0.04% | G | Yes | 11.5% energy sector emissions reductions; energy target | By 2030 | Below 2030 |
| Papua New Guinea | 0.09% | G | Yes | carbon neutrality target within the energy industries sub-sector by 2030, and a reduction target in annual emission from deforestation and forest degradation | By 2030 | - |
| Paraguay | 0.16% | B | Yes (++) | 10% (conditional: 20%) | By 2030 | Below BAU |
| Peru | 0.34% | F | Yes (++) | Fixed level target: 208.8 MtCO_2_eq (unconditional) and 179.0 MtCO_2_eq (conditional) | By 2030 | Fixed target |
| Philippines | 0.46% | C | Yes | 2% (Conditional: 75%) | By 2030 | Below BAU |
| Qatar | 0.32% | G | Yes | Conditional: 25% | By 2030 | Below BAU |
| Republic of Korea | 1.23% | A | Yes (++) | 40% | By 2030 | Below 2018 |
| Republic of Moldova | 0.15% | A | Yes (++) | 70% (conditional: 88%) | By 2030 | Below 1990 |
| Russian Federation | 3.68% | A | Yes (++) | 30% | By 2030 | Below 1990 |
| Rwanda | 0.02% | G | Yes | Mitigation actions only | by 2030 |  |
| Saint Kitts and Nevis | <0.01% | B | Yes (++) | 61% | By 2030 | Below 2010 |
| Saint Lucia | <0.01% | G | Yes (++) | 7% GHG emissions reduction in the energy sector | By 2030 | Below 2010 |
| Saint Vincent and Grenadines | <0.01% | B |  | 22% | By 2030 | Below BAU |
| Samoa | <0.01% | G | Yes | No explicit target for emission reduction | - | - |
| San Marino | <0.01% | A |  | 20% | By 2030 | Below 2005 |
| Sao Tome and Principe | <0.01% | B | Yes | Conditional: 24% | by 2030 | below 2005 |
| Saudi Arabia | 1.34% | C |  | List of policies and measures, leading to reduce emissions by 130 MtCO_2_eq by 2030 | By 2030 | below BAU |
| Senegal | 0.09% | C | Yes (++) | 7% by 2030 (conditional: 30%) | by 2030 | below BAU |
| Serbia | 0.18% | A | Yes | 9.8% | By 2030 | Below 1990 |
| Seychelles | <0.01% | B | Yes | 29% (21.4%) | By 2030 (2025) | Below BAU |
| Sierra Leone | 0.02% |  | Yes | No explicit target for emission reduction | - | - |
| Singapore | 0.11% | D | Yes | Reduce emission intensity by 36%, emissions peaking around 2030 | By 2030 | Below 2005 |
| Solomon Islands | 0.08% | A | Yes (++) | 47% (33%) | By 2030 (2025) | Below BAU |
| Somalia | 0.08% |  | Yes | 30% | By 2030 | - |
| South Africa | 1.13% | B |  | By 2025 and 2030, emissions will be in a range between 398 and 614 MtCO_2_eq, peaking between 2020 and 2025 | By 2030 | Below BAU |
| South Sudan | <0.01% | G |  | List of policies and measures | - | - |
| Sri Lanka | 0.06% | E | Yes (++) | 4% (conditional: 14.5%) | By 2030 | Below BAU |
| Sudan | 0.33% | G | Yes | List of policies and measures | By 2030 | - |
| Suriname | <0.01% | G | Yes | Mitigation actions only | By 2015 | N.A. |
| Swaziland | 0.01% | G |  | No explicit target for emission reduction | By 2030 | - |
| Switzerland | 0.09% | A | Yes (++) | At least 50% | By 2030 | Below 1990 |
| Taiwan | 0.55% | B |  | 50% | By 2030 | Below BAU |
| Tajikistan | 0.04% | A | Yes | not exceed 60-70% (unconditional), and 50-60% (conditional) of 1990 emissions levels by 2030. | - | - |
| Thailand | 0.77% | B | Yes | 20% (conditional: 25%) | By 2030 | Below BAU |
| Togo | 0.04% | B |  | 20% (conditional: 30%) | By 2030 | Below BAU |
| Tonga | <0.01% |  | Yes | List of energy goals | By 2030 | - |
| Trinidad and Tobago | 0.09% | B |  | 30% in public transport; plus conditional 15% in power generation, transport and industrial sectors | By 2030 | Below BAU |
| Tunisia | 0.07% | D | Yes (++) | 18% decrease in carbon intensity (conditional: 45%) | By 2030 | Below 2010 |
| Turkey | 1.1% | B |  | 21% | By 2030 | Below BAU |
| Turk-menistan | 0.25% | E |  | No explicit target for emission reduction | - | - |
| Tuvalu | <0.01% |  |  | Indicative: 60% | By 2025 | Below 2010 |
| Uganda | 0.12% | B |  | Mitigation actions only: The estimated potential impact could be 22% | By 2030 | Below BAU |
| Ukraine | 0.50% | A | Yes (++) | 65% | By 2030 | Below 1990 |
| United Arab Emirates | 0.59% | E | Yes | List of policies and measures, including an increase of renewable energy to 24% of the total energy mix by 2021 | By 2021 and 2030 | Below BAU |
| United Kingdom | 0.92% | A | Yes (++) | 68% reduction in GHG emissions in 2030 from 1990 levels | By 2030 | Below 1990 |
| United Republic of Tanzania | 0.3% | B | Yes | 30% (conditional: 35%) | By 2030 | Below BAU |
| United States of America | 11.75% | A | Yes (++) | 50% to 52% | By 2030 | Below 2005 |
| Uruguay | 0.07% | E |  | A list of sectorial targets sorted by GHG gas | - | - |
| Vanuatu | <0.01% | G | Yes (++) | Conditional: new reduction targets in energy sector (100% renewables for electricity) | By 2030 | Below BAU |
| Venezuela | 0.46% | C |  | 20% | By 2030 | Below BAU |
| Viet Nam | 0.79% | B | Yes (++) | 9% (conditional: 27%) | By 2030 | Below BAU |
| Yemen | 0.05% | B |  | 1% (conditional: 14%) | By 2030 | Below BAU |
| Zambia | 0.18% | E | Yes | 25% (conditional: 47%) | by 2030 | below 2010 |
| Zimbabwe | 0.22% | E |  | Conditional: 33% | by 2030 | below BAU |
| Total share | **97.4%** |  |  |  |  |  |

1. Source: GHG emissions in 2019 including emissions and removals from LULUCF based on the EDGAR database ([Olivier and Peters, 2020](#_ENREF_11)) (GHG emissions excluding those from LULUCF) and FAOSTAT ([2020](#_ENREF_5)) (LULUCF emissions);
2. Source: Climate Watch ([2022](#_ENREF_2))
3. Source: Climate Watch ([2022](#_ENREF_2))

# Supplementary Text 3: Methodology for the current policies scenario

The impact of the most effective current and planned policies on greenhouse gas emissions up to 2030 has been projected by the NewClimate Institute, IIASA and PBL ([den Elzen et al., 2016](#_ENREF_3); [den Elzen et al., 2019](#_ENREF_4); [Kuramochi et al., 2019](#_ENREF_8); [Kuramochi et al., 2021](#_ENREF_9)). Current and planned policies were selected on the basis of literature research and expert knowledge. Experts involved in the CD-LINKS project ([www.cd-links.org](http://www.cd-links.org/)) and the ENGAGE project (<https://www.engage-climate.org/>) reviewed the full list of policies, after which a shortlist was compiled of those expected to have the greatest impact on greenhouse gas emissions, for further quantification. The calculations by NewClimate Institute were largely based on its analyses for the Climate Action Tracker project, jointly carried out with Climate Analytics ([Climate Action Tracker, 2021](#_ENREF_1)). For the calculation, existing scenarios from national and international studies were used, as well as NewClimate Institute’s own calculations of the impact of individual policies in various sub-sectors. PBL based their calculations for the current policies scenarios (all sectors, except land use) on the IMAGE model; IIASA’s projections (the land-use sector only) were based on their global land-use model GLOBIOM and global forest model G4M. Basis of the PBL calculations of the impact of climate policies is the SSP2 baseline (no climate policy), as implemented in the IMAGE model ([Roelfsema et al., 2020](#_ENREF_13); [Van Vuuren et al., 2017](#_ENREF_19)) . Current climate and energy policies in G20 countries, as identified in the CD-LINKS project and ENAGE project, were added to that baseline ([Roelfsema et al., 2022](#_ENREF_12); [Roelfsema et al., 2020](#_ENREF_13); [van Soest et al., 2021](#_ENREF_18)).

For countries that are part of a larger IMAGE region (Australia, Kazakhstan, Republic of Korea, Russian Federation, and Ukraine), emissions projections were downscaled using the country’s share in the region’s 2015 emissions as a constant scaling factor.

**Land-use change accounting method**: Most of the 25 analysed countries and many of the remaining countries report emission target levels that include CO_2_ removals from activities related to the land use, land use change and forestry (LULUCF) sector. Although there are uncertainties concerning which accounting approaches and methodologies countries will apply to account for LULUCF related emissions and removals, we assume that a majority of countries will apply the net-net accounting approach, so land use emissions are included in the same way as the emissions from the other sectors (see Supplementary Text 4).

The GHG emissions projections under current policies were harmonised to the latest historical emissions data described below, i.e. adding the absolute emissions difference in the harmonisation year between the inventory data and the model data to the model projections, based on Nascimento et al. ([2021](#_ENREF_10)). The harmonisation year was updated to 2019 for Annex I countries and the latest data year for non-Annex I countries.

**Historical emissions**: For Annex I countries (Australia, Canada, the European Union, Japan, Kazakhstan, the Russian Federation, Turkey, the USA and Ukraine), the historical emissions data is based on the Greenhouse Gas Inventories submitted in 2021 to the UNFCCC ([UNFCCC, 2021a](#_ENREF_14)). For many non-Annex I countries, the historical data was taken from the national reports (National Communications and Biennial Update Reports) ([UNFCCC, 2021a](#_ENREF_14), [b](#_ENREF_15), [c](#_ENREF_16)) (for details for the 25 major emitting countries, see Nascimento et al. ([2021](#_ENREF_10))). For China, LULUCF projections are harmonized to the historical data from the 2014 GHG inventories presented in the Second Biennial Update Report (BUR2) of China ([Government of China, 2018](#_ENREF_7)). In the BUR2 report, the LULUCF sink was reported as -1115 MtCO_2_eq for 2014.

All GHG emission figures are expressed using the 100-year global warming potentials (GWPs) from the Intergovernmental Panel on Climate Change (IPCC) Fourth Assessment Report.

# Supplementary Text 4: Projected greenhouse gas emission targets levels from the NDCs of the G20 economies by 2030

Table S.2. Comparison of the unconditional and conditional mitigation targets for 2030, stated in the updated NDCs and first NDCs of the G20 countries. The calculation of the first NDC are based on earlier studies ([den Elzen et al., 2016](#_ENREF_3); [Kuramochi et al., 2019](#_ENREF_8)).

| G20 member | Unconditional NDC target (Conditional NDC target) in 2030 | | base year/emission level (MtCO_2_eq) | | Emission target (MtCO_2_eq) in 2030 | |
| --- | --- | --- | --- | --- | --- | --- |
|  | **First NDC** | **Updated NDC** | **First NDC** | **Updated NDC** | **First NDC** | **Updated NDC** |
| Argentina | -15% (-30%) | 26% | 2030 (592) | 483 | 483 (369) | 349 |
| Australia | -26% to -28% | -26% to -28%* | 2005 (601) | 2005 (612) | 433 to 445 | 440 to 452 |
| Brazil | -43% | -43% | 2005 (2,100) | 2005 (2,400 to 2,800)^1^ | 1,200 | 1,370 to 1,600^1^ |
| Canada | -30% | At least 40-45% | 2005 (749) | 2005 (739) | 524 | 371 to 408^2^ |
| China | CO_2_ emission peak 2030;  60-65% CO_2_ intensity reduction; 20% non-fossil fuels in energy; carbon sink | CO_2_ emission peak before 2030; 65% CO_2_ intensity reduction; 25% non-fossil fuels in energy; carbon sink | 2005 (7,050) | 2005 (7,050) | 14,483^2^  [12,900; 15,775] ^3^ | 13,745 [12,010; 14,530] ^3^ |
| EU27 | -40% | At least -55% | 1990 (5,446) for EU-28 | 1990 (4,633) | 3,243 | 2,085 |
| India | 33% to 35% emissions intensity reduction; 40% non-fossil fuel electricity; carbon sink | − | 2005 (1,650) | − | 4,445 [4,378; 5,091]  (4,170  [4,170; 4,466]) ^3^ |  |
| Indonesia | -29% (-41%) | -29% (-41%) | 2030 (2,869) | 2030 (2,869) | 2,037 (1,693) | 2,037 (1,693) |
| Japan | -26% | -46% | Fiscal Year 2013 (1,408) | Fiscal Year 2013 (1,408) | 1,042 | 807 ^5^ |
| Mexico | -22% (-36%) | − | 2030 (973) | 2030 (991) | 759 (623) | 773 (634) |
| Russian Federation | -25% to -30% | -30% | 1990 (3,087) | 1990 (3,087) | 2,315 to 2,161 | 2,161 |
| South Africa | Range 398 to 614 | Range 350 to 420 | 2030 (-) | − | 398 to 614 | 350 to 420 |
| Republic of Korea | -37% | -35% | 2030 (851) | 2018 (728) | 536 | 437 |
| Turkey | -21% | − | 2030 (1,175) | − | 928 | − |
| United Kingdon | -40% (as part of EU-28) | -68% | 758 | 758 | 435 | 260 |
| United States | -26% to -28% (2025) | -50% to -52% (2030) | 2005 (6,439) | 6,635 | 4,636 to 4,765 | 3,185 to 3,317 |

* Same as first NDC

^1^ There is an argument for the use of the 2005 emissions of the Third National Communication, as the new NDC refers to “Third National Communication from Brazil to the United Nations Framework Convention on Climate Change”, submitted on 20 April 2016.". The Third National Communication and Biennial Update Report (BUR) gives a 2005 emissions of 2700 MtCO_2_eq for GWP SAR, as can be on page 45 (BUR). For this analysis this estimate is corrected for GWP AR4, leading a base-year emissions of about 2800 MtCO_2_eq, which is also confirmed in the BUR. On the other hand, the NDC also specifies that “Information on emissions in 2005 and reference values may be updated and recalculated due to methodological improvements applicable to the inventories.” The Fourth National Communication would definitely fall under the category, thus the 2005 estimate from the 4^th^ NC would be updating the estimate from the 3^rd^ NC. See Table below.

| GHG emissions (GtCO_2_eq) | 2005 (base year) | 2025 | 2030 |
| --- | --- | --- | --- |
| 1^st^ NDC (2015) | 2.1 | 1.3 | 1.2 |
| 2^nd^ NDC (2020) based on 3^rd^ NatCom | 2.8 | 1.8 | 1.6 |
| 2^nd^ NDC (2020) based on 4^th^ NatCom | 2.4 | 1.5 | 1.4 |

^2^ Including land use credits

^3^ Central estimate based on this study, and ranges are based on Nascimento et al. ([2021](#_ENREF_10)).The intensity target for India is assumed to apply to total GHG emissions excluding agricultural and land use emissions Although the NDC of India did not specify to what GHG emission categories the intensity target applies, agricultural and land use emissions were excluded conform India’s reduction proposal for 2020.

^4^ A land use accounting approach is specified as Kyoto Protocol approach (gross-net accounting). A reduction of net LULUCF emissions is expected in the range of 47.7 MtCO_2_eq.

# Supplementary Text 5: LULUCF accounting method chosen for quantification

Table S.3 provides an overview of how the NDC targets have been quantified for the G20 countries and lists them by the accounting method which we have assumed. Most of the analysed countries report emission target levels that include emissions and removals from activities related to the LULUCF sector. Although there are uncertainties concerning which accounting approaches and methodologies countries will apply to account for LULUCF related emissions and removals towards their NDC reduction targets, we assume that a majority of countries will apply the net-net accounting approach ([den Elzen et al., 2016](#_ENREF_3)).3F^^[[1]](#footnote-2)^^ When applying this approach, all emissions and removals from the LULUCF sector are accounted for when calculating this base year estimate and target year estimate. In other words, the sector is treated like any other sector that is included in the NDC target.

A few countries have established accounting approaches for each LULUCF sub-sector and documented these in national documents and regulations. Two such examples are Canada^[[2]](#footnote-3)^ and the EU27^[[3]](#footnote-4)^. For the EU27 we apply a simplistic approach and assume that the EU27 will account for all LULUCF sub-sectors using the net-net accounting approach as this is the case for the majority of the sub-sectors. For Canada, LULUCF emissions are excluded for the base year when calculating the NDC target in terms of MtCO_2_ eq in 2030. However, the LULUCF sector does based on the selected accounting approach provide a set of carbon credits or debits when assessing if Canada has reached their NDC target. This calculation of the amount of credits varies between the sub-sectors but overall compares the LULUCF emissions in 2005 to the net emission in 2030 (i.e. a net-net estimate). For this assessment we make a simple assumption assume that Canada will be using the net-net accounting for all sub-sectors.

For countries that explicitly mentioned in their NDCs that emissions and removals from the LULUCF sector are excluded (Saudi Arabia), the LULUCF sector is excluded from the calculation of the NDC target emission levels and current policies scenario projections.

An example of a country that applies a different approach is that of Republic of Korea that explicitly state in their NDCs that emissions and removals from the LULUCF sector are excluded when calculating their emissions for the base year (2017), but that emissions and removals from the LULUCF are to be accounted for when assessing their target of total GHG emission reduction by 2030. As the LULUCF sector was a sink of -44.5 MtonCO_2_eq in 2017 ([UNFCCC, 2021b](#_ENREF_15)), this selective accounting approach facilitates Korea’s efforts to reduce their domestic emission.

Further discussions concerning how individual countries consider the LULUCF sector within their previous NDCs can be found in Forsell et al. ([2016](#_ENREF_6)). Russia, India, and US are all assumed to treat the LULUCF sector in the same way for this assessment as no additional specifications has yet been provided by these countries.

Table S.3. Overview of NDC configuration for the G20 countries

| Country | Target type | LULUCF sector is included in the NDC | Approach for NDC quantification by authors | | |
| --- | --- | --- | --- | --- | --- |
|  |  |  | Reference emissions include LULUCF | LULUCF treated separately | LULUCF accounting method applied |
| Argentina | Baseline specified | Yes | Yes | No | Net-Net |
| Australia | Base year | Yes | Yes | No | Net-Net |
| Brazil | Base year | Yes | Yes | No | Net-Net |
| Canada | Base year | Yes | No | Yes | Net-Net |
| China | Intensity and non-GHG | Yes | Yes | No | Net-Net |
| EU27 | Base year | Yes | Yes | No | Net-Net |
| India | Intensity and non-GHG | Yes | Yes | No | Net-Net |
| Indonesia | Baseline specified | Yes | Yes | No | Net-Net |
| Japan | Base year | Yes | Yes | No | Gross-Net |
| Mexico | Baseline specified | Yes | Yes | No | Net-Net |
| Republic of Korea | Baseline specified | Yes | Yes | No | Gross-Net |
| Russian Federation | Base year | Yes | Yes | No | Net-Net |
| South Africa | Trajectory | Yes | Yes | No | Net-Net |
| Turkey | Baseline specified | Yes | Yes | No | Net-Net |
| USA | Base year | Yes | Yes | No | Net-Net |
| Saudi Arabia | Trajectory | No | No | No | None |

# Supplementary Text 6: Projected global greenhouse gas emission levels by 2030

Table S.4: Greenhouse gas emissions (including LULUCF) in G20 economies, selected non-G20 countries and global emission levels, projected for 2030 for the IMAGE current policies scenario, the unconditional NDC scenario and the conditional NDC scenario.

| GHG emissions (MtCO_2_eq) |  |  | Current policies scenario | | | Unconditional NDC  scenario | | | Conditional NDC  scenario | | |
| --- | --- | --- | --- | --- | --- | --- | --- | --- | --- | --- | --- |
|  | **2005** | **2015** | **2030** | **min** | **max** | **2030** | **min** | **max** | **2030** | **min** | **max** |
| Argentina | 418 | 379 | 349 | 349 | 349 | 357 | 357 | 357 | 349 | 349 | 349 |
| Australia | 612 | 534 | 541 | 466 | 541 | 447 | 441 | 452 | 447 | 441 | 452 |
| Brazil | 2178 | 1373 | 1806 | 1183 | 1806 | 1511 | 1370 | 1600 | 1511 | 1370 | 1600 |
| Canada | 739 | 723 | 688 | 681 | 688 | 390 | 371 | 408 | 390 | 371 | 408 |
| China | 7150 | 11692 | 14530 | 12111 | 14530 | 13745 | 12010 | 14530 | 13745 | 12010 | 14530 |
| EU27 | 4225 | 3516 | 2197 | 2197 | 2861 | 2085 | 2085 | 2085 | 2085 | 2085 | 2085 |
| India | 1650 | 2426 | 4170 | 4170 | 4466 | 5010 | 4929 | 5091 | 4445 | 4378 | 5091 |
| Indonesia | 1317 | 2404 | 2074 | 1886 | 2105 | 2037 | 2037 | 2037 | 1693 | 1693 | 1693 |
| Japan | 1289 | 1262 | 1017 | 863 | 1017 | 807 | 807 | 807 | 807 | 807 | 807 |
| Mexico | 605 | 724 | 774 | 759 | 836 | 773 | 773 | 773 | 634 | 634 | 634 |
| Russia | 1435 | 1447 | 1722 | 1345 | 1722 | 2161 | 2161 | 2161 | 1647 | 1647 | 1647 |
| Saudi Arabia | 417 | 640 | 984 | 933 | 1035 | 1034 | 854 | 1214 | 1034 | 854 | 1214 |
| South Africa | 497 | 550 | 472 | 432 | 472 | 385 | 350 | 420 | 385 | 350 | 420 |
| South Korea | 510 | 654 | 547 | 547 | 698 | 437 | 437 | 437 | 437 | 437 | 437 |
| Turkey | 263 | 376 | 446 | 446 | 594 | 928 | 928 | 928 | 928 | 928 | 928 |
| United Kingdom | 698 | 515 | 383 | 383 | 383 | 260 | 260 | 260 | 260 | 260 | 260 |
| United States | 6635 | 5907 | 4900 | 4900 | 5492 | 3251 | 3185 | 3317 | 3251 | 3185 | 3317 |
| G20 economies | 30,636 | 35,122 | 36,953 | 32,851 | 38,736 | 35,045 | 32,819 | 36,896 | 33,773 | 31,615 | 35,275 |
| Chile | 84 |  | 124 | 122 | 126 | 95 | 95 | 95 | 95 | 95 | 95 |
| Colombia | 245 |  | 296 | 283 | 309 | 169 | 169 | 169 | 169 | 169 | 169 |
| DR Congo | 211 |  | 370 | 361 | 378 |  |  |  | 340 | 340 | 340 |
| Ethiopia | 144 |  | 194 | 162 | 226 | 348 | 348 | 348 | 126 | 126 | 126 |
| Kazakhstan | 231 |  | 426 | 396 | 438 | 315 | 315 | 315 | 278 | 278 | 278 |
| Morocco | 62 |  | 100 | 78 | 121 | 116 | 116 | 116 | 78 | 78 | 78 |
| Philippines | 155 |  | 287 | 287 | 287 |  |  |  | 96 | 102 | 90 |
| Thailand | 305 |  | 481 | 481 | 481 | 444 | 444 | 444 | 416 | 416 | 416 |
| Ukraine | 408 |  | 405 | 384 | 414 | 309 | 309 | 309 | 309 | 309 | 309 |
| Non-G20 countries | 12,540 |  | 17,028 | 19,471 | 19,471 | 17,419 | 17,253 | 18,354 | 18,853 | 21,296 | 21,296 |
| International bunker emissions | 1,035 | 1,184 | 1,699 | 1,699 | 1,699 | 1,699 | 1,699 | 1,699 | 1,699 | 1,699 | 1,699 |
| Remaining LULUCF CO_2_ emissions | 886 | 678 | 744 | 744 | 744 | 744 | 744 | 744 | 744 | 744 | 744 |
| Global, excluding the impact of overachievement | **45,153** | **51,181** | **56,826** | **52,592** | **59,552** | **56,341** | **54,115** | **58,193** | **53,244** | **51,086** | **54,746** |
| Additional reduction due to overachievement of NDCs* |  |  |  |  |  | 4057 | 4057 | 4057 | 2544 | 2544 | 2544 |
| Global emissions, including the impact of overachievement of NDCs * |  |  | **56,826** | **52,592** | **59,552** | **52,284** | **50,059** | **54,136** | **50,700** | **48,542** | **52,202** |

* In our assessment, we assumed that countries will follow their current policies emission trajectory if they overachieve their NDC targets, leading to additional net reductions at the global level of 4.6 and 2.8 GtCO_2_eq for the unconditional and conditional NDC scenario, respectively.

# Supplementary Text 7: GHG reduction targets for the G20 economies of the NDC including and excluding the LULUCF sector

As the NDC emission projections for the LULUCF sector for most G20 countries start as of 2015, NDC targets for the G20 countries based on their original base year were compared with NDC targets based on 2015 levels of GHG emission and removals (See table S.5). The NDC emission projections for the LULUCF sector could thus be harmonized to 2015 GHG emissions and removals without influencing the NDC reduction targets. LULUCF emissions and removals for 2015 used for the harmonization are shown in Table S.5 as well as resulting LULUCF emission and removals for the resulting 2030 NDC emission projections for the LULUCF sector. The development for the LULUCF sector has been estimated by the GLOBIOM/G4M model.

Table S.5: Calculation of the NDC reduction targets for the G20 countries harmonized to 2015 values and harmonized depending on the country selection to include or exclude LULUCF emissions and removals.

**Reduction targets of the NDCs (incl. LULUCF and excl. LULUCF)**

|  | Compared to Base-year target (2005, or 1990)* | Compared to 2015 (incl. LULUCF) | Compared to 2015, excl. LULUCF | LULUCF emissions (2015) (MtCO_2_eq) | LULUCF emissions of NDCs (2030) (MtCO_2_eq) |
| --- | --- | --- | --- | --- | --- |
| Argentina | -14% | -6% | -3% | 34 | 22 |
| Australia | -27% | -16% | -16% | 1 | -2 |
| Brazil | -31% | 10% | 9% | 184 | 216 |
| Canada | -47% | -46% | -43% | 4 | -22 |
| China | 92% | 18% | 12% | -1123 | -653 |
| EU27 | -55%* | -41% | -37% | -292 | -300 |
| India | 169% | 83% | 75% | -296 | -306 |
| Indonesia | 55% | -15% | 126% | 1569 | 151 |
| Japan | -37% | -36% | -32% | -57 | -93 |
| Mexico | 28% | 7% | 13% | 19 | -22 |
| Republic of Korea | -23% | -37% | -33% | -43 | -56 |
| Russia | -30%* | 49% | 28% | -589 | -451 |
| Saudi Arabia | 148% | 61% | 61% | -9 | -9 |
| South Africa | -22% | -30% | -30% | -28 | -23 |
| Turkey | 253% | 147% | 118% | -97 | -105 |
| United Kingdom | -68% | -49% | -50% | 6 | 6 |
| United States | -51% | -45% | -42% | -764 | -630 |
| Argentina | -14% | -6% | -3% | 34 | 22 |
| G20 | 16% | 1% | 3% | -1774 | -2577 |

*Only for the EU and Russian Federation 1990

# Supplementary Figure 1: The contribution of the updated NDC targets of countries on total additional global emissions reduction


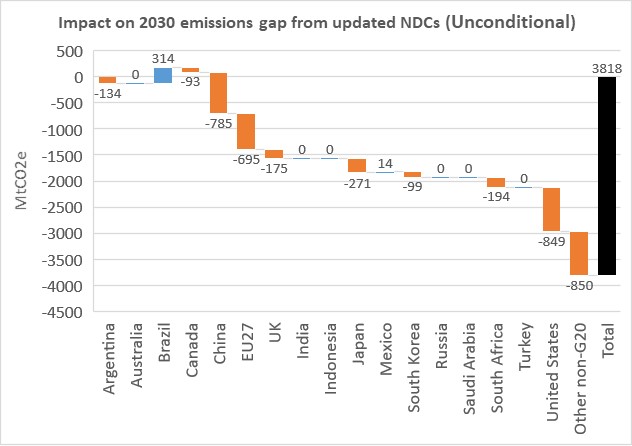

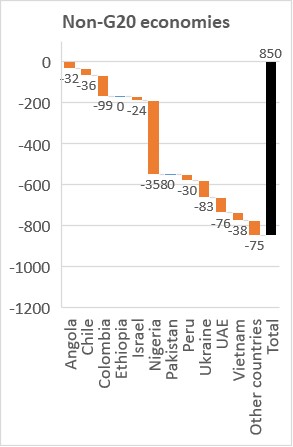


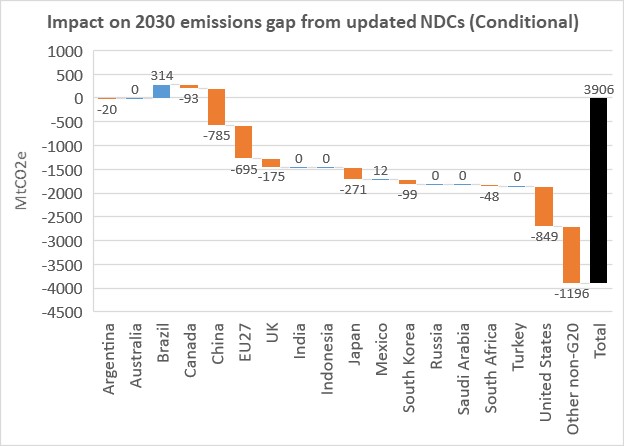

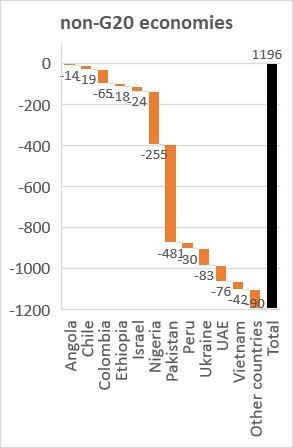


Figure S.2. The contribution of the updated NDC targets of countries on total additional global emissions reduction (as indicated in black column) based on the full implementation of the conditional and unconditional NDC targets (upper and lower figure), compared to the previous NDCs. The red columns indicate additional reductions from countries with their stronger NDC targets (compared to their previous NDCs), and the blue columns indicate the increase in emissions from countries with their weaker NDC targets.

# References

Climate Action Tracker (2021) Country Assessments Climate Action Tracker (updated September 2021) (WWW document) (Climate Analytics, NewClimate Institute). URL: <https://climateactiontracker.org/countries/> (Accessed: 18 September 2021).

ClimateWatch (2022) Explore Nationally Determined Contributions (NDCs). World Resource Institute, <https://www.climatewatchdata.org/ndcs-explore> (accessed 31 Janauary 2022), Washington DC, USA.

den Elzen M, Admiraal A, Roelfsema M, van Soest H, Hof AF, Forsell N (2016) Contribution of the G20 economies to the global impact of the Paris agreement climate proposals. Climatic Change 137:655–665 <https://doi.org/10.1007/s10584-016-1700-7>.

den Elzen MGJ, Kuramochi T, Höhne N, Cantzler J, Esmeijer K, Fekete H, . . . Vandyck T (2019) Are the G20 economies making enough progress to meet their NDC targets? Energy Policy 126:238-250 <https://doi.org/10.1016/j.enpol.2018.11.027>.

FAOSTAT (2020) Land use emissions. Food and Agricultural Organization of the United Nations (FAO), Rome, Italy, <http://faostat3.fao.org/download/G2/*/E>.

Forsell N, Turkovska O, Gusti M, Obersteiner M, Den Elzen M, Havlik P (2016) Assessing the INDCs' land use, land use change, and forest emission projections. Carbon Balance and Management 11, 26, <https://doi.org/10.1186/s13021-016-0068-3>.

Government of China (2018) The People’s Republic of China Second Biennial Update Report on Climate Change. <https://www4.unfccc.int/sites/SubmissionsStaging/NationalReports/Documents/23146085_China-BUR2-1-China_BUR2_English.pdf>.

Kuramochi T, Nascimento L, de Villafranca Casas MJ, Fekete H, de Vivero G, Lui S, . . . Gusti M (2019) Greenhouse gas mitigation scenarios for major emitting countries. Analysis of current climate policies and mitigation commitments: 2019 update, NewClimate Institute (Cologne, Germany), PBL (The Hague, the Netherlands), IIASA (Laxenburg, Austria), <https://www.pbl.nl/en/publications/greenhouse-gas-mitigation-scenarios-for-major-emitting-countries-2019-update>.

Kuramochi T, Nascimento L, Moisio M, den Elzen M, Forsell N, van Soest H, . . . Höhne N (2021) Greenhouse gas emission scenarios in nine key non-G20 countries: An assessment of progress toward 2030 climate targets. Environmental Science & Policy 123:67-81 <https://doi.org/10.1016/j.envsci.2021.04.015>.

Nascimento L, Kuramochi T, de Villafranca Casas MJ, Fekete H, de Vivero G, Lui S, . . . Gusti M (2021) Greenhouse gas mitigation scenarios for major emitting countries. Analysis of current climate policies and mitigation commitments: 2021 update, NewClimate Institute (Cologne, Germany), PBL (The Hague, the Netherlands), IIASA (Austria), forthcoming, <https://newclimate.org/2021/10/07/ghg-mitigation-scenarios-for-major-emitting-countries-analysis-of-current-climate-policies-2021-update/>.

Olivier JGJ, Peters JAHW (2020) Trends in global emissions of CO2 and total greenhouse gases: 2020 Report. PBL Netherlands Environmental Assessment Agency, The Hague, the Netherlands, <https://www.pbl.nl/en/publications/trends-in-global-co2-and-total-greenhouse-gas-emissions-2020-report>.

Roelfsema M, van Soest HL, den Elzen M, de Coninck H, Kuramochi T, Harmsen M, . . . van Vuuren DP (2022) Developing scenarios in the context of the Paris Agreement and application in the integrated assessment model IMAGE: a framework for bridging the policy-modelling divide. Environmental Science and Policy 135:104-116 <https://doi.org/10.1016/j.envsci.2022.05.001>.

Roelfsema M, van Soest HL, Harmsen M, van Vuuren DP, Bertram C, den Elzen M, . . . Vishwanathan SS (2020) Taking stock of national climate policies to evaluate implementation of the Paris Agreement. Nature Communications 11:2096 <https://doi.org/10.1038/s41467-020-15414-6>.

UNFCCC (2021a) Greenhouse Gas Inventory Data - Detailed data by Party, <http://di.unfccc.int/detailed_data_by_party>

UNFCCC (2021b) Submitted Biennial Update Reports (BURs) from Non-Annex I Parties, <https://unfccc.int/BURs>.

UNFCCC (2021c) Submitted National Communications from Non-Annex I Parties, <https://unfccc.int/non-annex-I-NCs>.

UNFCCC (2022) NDC registry, <https://www4.unfccc.int/sites/NDCStaging/Pages/LatestSubmissions.aspx> (Accessed: 30 January 2022).

van Soest HL, Aleluia Reis L, Baptista LB, Bertram C, Després J, Drouet L, . . . van Vuuren DP (2021) Global roll-out of comprehensive policy measures may aid in bridging emissions gap. Nature Communications 12, 6419, <https://doi.org/10.1038/s41467-021-26595-z>.

Van Vuuren DP, Stehfest E, Gernaat DE, Doelman JC, Van den Berg M, Harmsen M, . . . Edelenbosch OY (2017) Energy, land-use and greenhouse gas emissions trajectories under a green growth paradigm. Global Environmental Change 42:237–250 <http://dx.doi.org/10.1016/j.enpol.2015.11.030>.

1. In this approach, activities are accounted using the reported net emissions in each year of the accounting period minus the net emissions in the base year. In the situation where the net emissions have decreased, a country may issue credits (i.e. removal units, or RMUs) and if net emissions have increased, it must cancel units (i.e. take on debits). The net-net LULUCF accounting method implies that credits and debits from the LULUCF sector are treated in the same way as any other GHG inventory sector, where emissions are compared to those in the base year. [↑](#footnote-ref-2)
2. For further information about the accounting approaches that have been established for Canada we refer to 2018 Canada’s Greenhouse Gas and Air Pollutant Emissions Projections (<http://publications.gc.ca/collections/collection_2018/eccc/En1-78-2018-eng.pdf>) and Canada’s Fourth Biennial Report on Climate Change (<https://unfccc.int/documents/209928> ). [↑](#footnote-ref-3)
3. For further information about the accounting approaches have been established for EU27 we refer to the Revision of the Regulation on the inclusion of greenhouse gas emissions and removals from land use, land use change and forestry (<https://ec.europa.eu/info/sites/default/files/revision-regulation-ghg-land-use-forestry_with-annex_en.pdf>) [↑](#footnote-ref-4)
